# A Unique SUMO-2-Interacting Motif within LANA Is Essential for KSHV Latency

**DOI:** 10.1371/journal.ppat.1003750

**Published:** 2013-11-21

**Authors:** Qiliang Cai, Shen Cai, Caixia Zhu, Suhbash C. Verma, Ji-Young Choi, Erle S. Robertson

**Affiliations:** 1 MOE & MOH Key Laboratory of Medical Molecular Virology, School of Basic Medical Science, Shanghai Medical College, Fudan University, Shanghai, People's Republic of China; 2 Department of Microbiology and Abramson Comprehensive Cancer Center, Perelman School of Medicine at the University of Pennsylvania, Philadelphia, United States of America; 3 Department of Microbiology and Immunology, School of Medicine University of Nevada, Reno, Nevada, United States of America; University of Southern California Keck School of Medicine, United States of America

## Abstract

Kaposi's sarcoma-associated herpesvirus (KSHV) stabilizes hypoxia-inducible factor α (HIF-1α) during latent infection, and HIF-1α reactivates lytic replication under hypoxic stress. However, the mechanism utilized by KSHV to block lytic reactivation with the accumulation of HIF-1α in latency remains unclear. Here, we report that LANA encoded by KSHV contains a unique SUMO-interacting motif (LANA^SIM^) which is specific for interaction with SUMO-2 and facilitates LANA SUMOylation at lysine 1140. Proteomic and co-immunoprecipitation analysis further reveal that the SUMO-2 modified transcription repressor KAP1 is a critical factor recruited by LANA^SIM^. Deletion of LANA^SIM^ led to functional loss of both LANA-mediated viral episome maintenance and lytic gene silencing. Moreover, hypoxia reduced KAP1 SUMOylation and resulted in dissociation of both KAP1 and Sin3A repressors from LANA^SIM^-associated complex. Therefore, the LANA^SIM^ motif plays an essential role in KSHV latency and is a potential drug target against KSHV-associated cancers.

## Author Summary

Hypoxia stress is a common feature of tumor microenvironment and is widely associated with pathogenesis linked to many oncogenic viruses. Kaposi's sarcoma-associated herpesvirus (KSHV), the etiological cause of Kaposi's sarcoma and primary effusion lymphoma, has been reported to encode several proteins that usurp hypoxia signaling during infection. One encoded protein LANA is a latent protein important for regulation of KSHV life cycle and cell transformation. The molecular mechanisms of how KSHV controls life cycle in normoxia and hypoxia is not fully understood. In this study, we show that LANA contains a unique SUMO-interacting motif (LANA^SIM^) which is specific for SUMO-2 binding. Importantly, SUMO-2 modified KAP1, a chromatin remodeling factor recruited by LANA^SIM^ is hypoxia sensitive, and plays a critical role in silencing viral gene expression. This discovery not only adds to our understanding of hypoxia-mediated remodeling of the viral episome in a SUMO dependent manner, but also provides a new dimension to development of therapeutic strategies against KSHV-associated cancers.

## Introduction

Kaposi's sarcoma-associated herpesvirus (KSHV), or human herpesvirus 8 (HHV-8), is the etiological agent of Kaposi's sarcoma (KS), and is tightly associated with primary effusion lymphoma (PEL) and a subset of multicentric Castleman's disease (MCD) [1], [2]. KSHV is a large DNA tumor virus encoding more than 90 open reading frames (ORF). Like all herpesviruses, KSHV has two distinct phases of its life cycle: latency and lytic replication. During latent infection, only a few genes are expressed [3]. The latency-associated nuclear antigen (LANA) encoded by ORF73 is one of the dominant latent proteins with multiple functions. These include tethering the viral episome to the host chromatin, inhibiting tumor suppressors, regulating gene transcription and blocking apoptosis for establishment and maintenance of latent infection (reviewed in [4], [5], [6]. The replication and transcriptional activator (RTA) encoded by ORF50 is essential for initiation of viral lytic replication [7], [8], [9], [10]. RTA drives lytic replication via activation of a transcription cascade of KSHV genes expressed during the early stages of the viral life cycle [7], [9]. Studies of KSHV-induced cell transformation *in vivo* demonstrated that KSHV predominantly enters a latent state [11], [12]. However, clinical analysis of KS patient samples have shown that a fraction of infected cells can also enter into lytic replication producing infectious virions and expressing virus-encoded paracrine signaling factors [13]. This suggests that spontaneous reactivation from latency in the tumor microenvironment is also important for growth and dissemination of viral infected tumor cells.

Hypoxic stress is a common feature of the tumor microenvironment [14], [15], [16], which results from oxygen consumption by successive layers of tumor cells distal to blood vessels or temporary vessel closure [17]. The extent of tumor hypoxia is strongly associated with tumor development and malignant progression. To elucidate the effect of intratumoral hypoxia on KSHV-associated cancers, we and other groups have shown that hypoxic stress reactivates KSHV lytic replication *in vitro* [18], [19]. In hypoxia, the major latent antigen LANA together with the key hypoxia responder HIF-1α (the inducible subunit of the heterodimeric transcriptional factor HIF-1) binds to the hypoxia-responsive elements (HRE, 5′-RCGTGC-3′) within the RTA gene promoter inducing lytic replication [18]. Interestingly, under normoxic conditions, HIF-1α is aberrantly accumulated in both KSHV latently infected cells and KS patient tissues in a LANA-dependent manner [20], [21]. These results indicate that LANA plays a dual role in controlling HIF-1α transcriptional activity for KSHV latent and lytic replication. However, the mechanism utilized by LANA to exert this dual function in normoxia and hypoxia remains unclear.

Emerging studies indicate that post-translational modification of proteins by the small ubiquitin-like modifier (SUMO) plays an important role in epigenetic control of gene transcription, and in response to hypoxic stress [22], [23], [24], [25]. SUMO is covalently attached to a lysine residue of target proteins through an isopeptide bond by three enzymes: heterodimeric SUMO-activating enzyme E1 (Uba2-Aos1), SUMO-conjugating enzyme E2 (Ubc9), and the substrate recognition factors or E3 ligases (i.e. PIAS protein family) [26]. Different from ubiquitination, SUMO conjugation often requires a consensus sequence ΨKxE/D (Ψ, large hydrophobic residue; x, any amino acid) around the target lysine [27]. The consequence of SUMOylation leads to various facets of protein function, including regulation of gene transcription, cell cycle, DNA repair and subcellular localization. In mammalian cells, there are at least four isoforms of SUMO – SUMO-1, -2, -3 and -4. SUMO-1 is the major SUMO in human cells; SUMO-2 and SUMO-3 are highly similar and responds to different cellular stresses [28], [29]; and SUMO-4 is tissue specific and so far only found in the pancreas [30]. In addition to attaching covalently to substrates, SUMO can also non-covalently bind to other proteins through a consensus SUMO-interacting motif (SIM), which is identified as sequence h-h-x-S-x-S/T-a-a [31], V/I-x-V/I-V/I [32], or K-x_3–5_-I/V-I/L-I/L-x_3_-D/E/Q/N-D/E-D/E [33]. Thus, in regards to the biological functions of these modified substrates, the SUMOylated targets may depend on their ability to interact with other effectors containing SIM motifs.

In this study, we demonstrate that LANA contains a unique SUMO-interacting motif (LANA^SIM^) specific for interaction with SUMO-2 and that LANA can also be SUMOylated on lysine 1140. Proteomic analysis further showed that LANA^SIM^ recruits a transcription inhibitory complex which includes two co-repressors KAP1 and Sin3A. Strikingly, we found that deletion of the LANA^SIM^ motif sufficiently abolishes its association with poly-SUMO2 modified KAP1, and loss of LANA's ability to support KSHV episome maintenance and gene silencing. Furthermore, hypoxia blocks poly-SUMO2 modified KAP1 and leads to dissociation of KAP1 and Sin3A from the LANA^SIM^ complex, as well as loss of LANA SUMOylation, which in turn transactivates viral gene expression for lytic replication. This describes the first mechanism by which a viral protein presents a specific SIM-motif platform to selectively recruit a cellular transcriptional complex for viral episome maintenance and gene silencing during latent infection.

## Results

### LANA contains a SIM motif which is specific for interaction with SUMO-2

**Figure 1:**
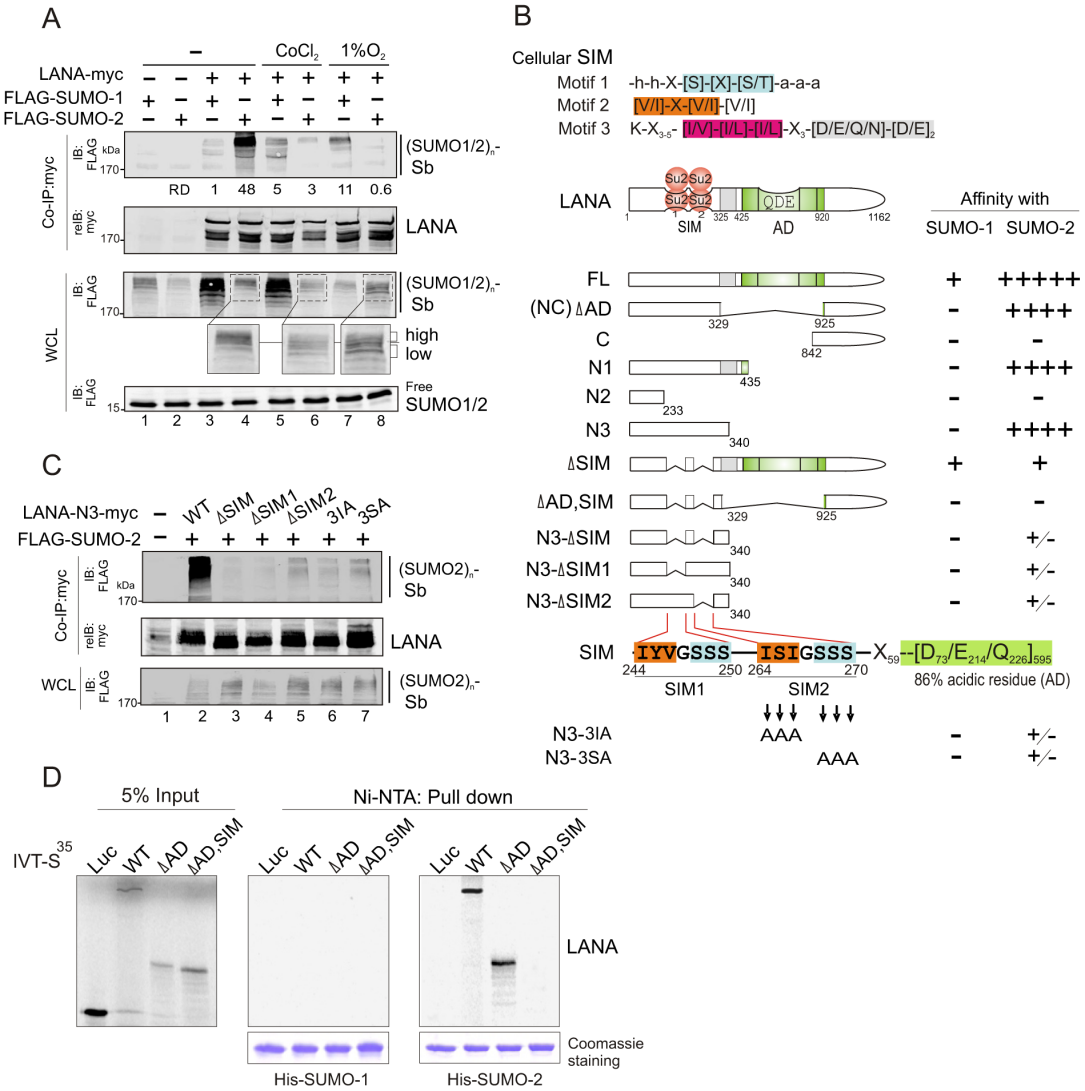
LANA contains a unique SUMO2-interacting motif. (**A**) LANA interacts with SUMO-2 and is sensitive to hypoxia. HEK293 cells were co-transfected with expression plasmids as indicated in the figure, and individually treated with or without hypoxia (100 µM CoCl_2_ or 1% O_2_) at 24 hr post-transfection for overnight before harvest. Cell extracts were subjected to co-immunoprecipitated (co-IP) and immunoblotting (IB) as indicated. The same membrane was stripped and reblotted (reIB) with indicated antibodies. The relative density (RD) of LANA-interacting SUMO-1 or SUMO-2 modified substrates [(SUMO1/2)n-sb] is shown. The position (>170 kDa) of hypoxia-sensitive SUMO-2 modified substrate [(SUMO2)n-sb] from whole cell lysate (WCL) is enlarged. The reduced level of (SUMO2)n-sb is highlighted as “high” and “low”. (**B**) Schematic representation of different LANA truncated mutants and the affinity of their interaction with SUMO-1/2. The relative affinity of LANA with SUMO-1 or SUMO-2 was summarized from *supplementary Figure S1* and *panel C*. The homology of SUMO-interacting motifs of LANA (LANA^SIM^: SIM1 and SIM2) with the cellular conserved SIM (motifs 1, 2 and 3) sequences, and residue positions of each LANA mutant is indicated. Su2, SUMO-2; SIM, SUMO-interacting motif; AD, acidic domain. (**C**) An intact SIM motif is required for LANA to interact with SUMO-2. HEK293 cells were co-transfected with expression plasmids as indicated. At 48 hr post-transfection, whole cell lysates (WCL) were subjected to co-immunoprecipitated (co-IP) and immunoblotting (IB) as indicated. The same membrane was stripped and reblotted (reIB) with indicated antibodies. (**D**) The SIM motif of LANA is required for interacting with SUMO-2 not SUMO-1 *in vitro*. *In vitro*-translated, radiolabeled wild type (WT) LANA and its mutants (ΔAD, ΔAD,SIM) was individually incubated with His-SUMO-1 or His-SUMO-2 recombinant proteins with Nickel-agarose (Ni-NTA) beads. His-SUMO-1 and His-SUMO-2 recombinant proteins are shown by Coomassie staining at the bottom panel. Bound complexes were analyzed by autoradiography. Luciferase (Luc) was used as negative control. Input was used at 5%.

To investigate whether LANA is involved with the SUMO-signaling pathway, we performed co-immunoprecipitation (co-IP) assays by co-expressing myc-tagged LANA with FLAG-tagged SUMO-1 or SUMO-2 under normoxic conditions. The results showed that LANA dramatically associated with the SUMO-2 not SUMO-1 modified substrate [(SUMO1/2)_n_-sb] with high molecular weight (>170 kDa) (Figure 1A, lanes 3 and 4). Interestingly, when hypoxic stress was induced by CoCl_2_ or 1% oxygen, the association of LANA with (SUMO2)_n_-sb was significantly reduced (Figure 1A, lanes 4, 6 and 8), while a moderate increase in the association of LANA with (SUMO1)_n_-sb was observed (Figure 1A, lanes 3, 5 and 7). In contrast, there was a reduced level of (SUMO2)_n_-sb [namely more “low” than “high” of (SUMO2)_n_-sb appearance] in whole cell lysates in hypoxia compared with normoxia (Figure 1A, compare lanes 4 with 6 and 8 in enlarged region), suggesting that the reduced association of LANA with (SUMO2)_n_-sb in response to hypoxia may be due to less SUMO-2 modification of target proteins.

To identify the domain required for LANA to interact with poly-SUMO-2, we performed similar co-IP assays by using different mutants of LANA. The results show that residues 233 to 340 at the amino terminus are critical for the strong interaction of LANA with the (SUMO2)_n_-sb (Figure 1B and supplementary Figure S1), and the central region which contains residues 329 to 925, a highly repetitive region provides minimal contribution to the interaction of LANA with (SUMO1)_n_-sb and (SUMO2)_n_-sb (Figure 1B and supplementary Figure S1C, upper panel). In contrast, as the position of (SUMO2)_n_-sb size did not change dramatically, even though the molecular weight of LANA truncated mutants (FL, NC, N3, N2 and N1) was varied from 65 kDa to 250 kDa. This indicates that the majority part of the detected (SUMO2)_n_-sb is not the SUMO2-modified LANA.

To further determine whether a cellular SUMO-interacting motif (SIM) -like sequence is located within the residues 233 to 340 of LANA, we aligned the LANA sequence with the three reported cellular SIM sequences [31], [32], [33]. The results identified two potential SIM-like motifs (SIM1 and SIM2) with partial homology to cellular SIM motifs both located within the residues 233 to 340 (Figure 1B and supplementary Table S1). To prove that these SIM motifs (LANA^SIM^) are required for LANA to interact with the (SUMO2)_n_-sb, we generated a series of mutations within the SIM motif of LANA 1–340 (N3) by site directed mutagenesis and again performed similar co-IP assays with SUMO-2. Strikingly, the results show that deletion of the SIM1, SIM2, SIM1/2, or mutation of three residues within SIM2 motif (3IA or 3SA) resulted in a significant reduction in the interaction of LANA 1–340 with (SUMO2)_n_-sb (Figure 1C). Therefore, we speculated that the intact 3D structure of the SIM motif within the amino terminus of LANA is important for interaction with the (SUMO2)_n_-sb. To verify whether the LANA^SIM^ motif is specifically required for LANA to preferentially associate with (SUMO2)_n_-sb instead of (SUMO1)_n_-sb, we performed similar assays using full-length LANA or its NC truncated mutant with or without deletion of the SIM motifs. Indeed, deletion of the LANA^SIM^ motif completely abrogated the interaction of the NC truncated mutant of LANA with (SUMO2)_n_-sb while no detectable effect with (SUMO1)_n_-sb (supplementary Figure S1B, lanes 3 and 6), and also specifically reduced the interaction of full length LANA with (SUMO2)_n_-sb but not (SUMO1)_n_-sb (supplementary Figure S1C, compare lanes 4, 5 with 2, 3). In addition, the results from *in vitro* pull-down assays of His-SUMO-1 or SUMO-2 recombinant protein with radioactive labeled wild type LANA or its SIM-deleted mutant showed that LANA only bound to SUMO-2 not SUMO-1 and this interaction of LANA with SUMO-2 was dependent on the LANA^SIM^ motif (Figure 1D), further confirming that the LANA^SIM^ motifs are critical for LANA to strongly interact with (SUMO2)_n_-sb through SUMO-2.

**Figure 2:**
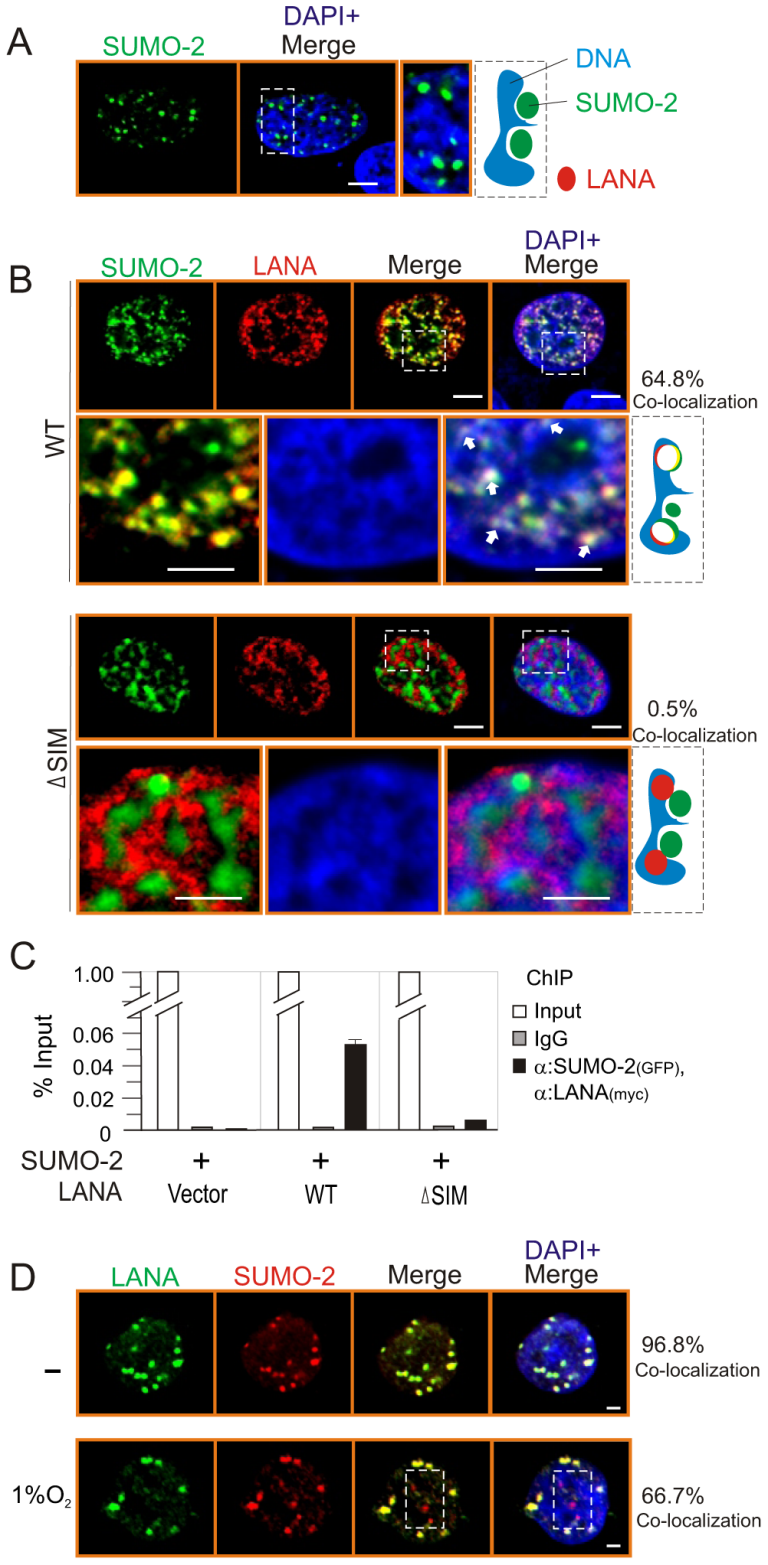
The LANA^SIM^ deletion reduces the co-localization of LANA with SUMO-2 on chromatin DNA. HEK293 cells co-transfected GFP-SUMO-2 with vector (**A**) alone, wild type (WT) or SIM-deleted (ΔSIM) mutant of LANA NC with myc tag (**B**) was individually cultured on coverslips, fixed with 3% paraformaldehyde, and then stained with anti-myc antibody as indicated. Bottom panels show the magnified view and arrowheads point examples of LANA and SUMO-2 co-localization on chromosome DNA within the outline region. Nuclei were counterstained with DAPI. Scale bars, 2 µm. Right panel shows schematic of co-localization (yield white) of LANA (red) and SUMO-2 (green) with host chromatin DNA (blue) and the percentage of co-localization from 50 counted cells. (**C**) Sequential ChIP was performed first with anti-GFP antibody or mouse IgG followed by a second ChIP with anti-myc antibody. Quantitative PCR was performed using TR specific primer. (**D**) Hypoxia reduces the co-localization of LANA with SUMO-2. BC3 cells were subjected to hypoxia (1% oxygen) treatment for overnight. Endogenous LANA and SUMO-2 were individually stained by LNA1 (rat) and SUMO-2 (rabbit) antibodies. SUMO-2 non-colocalization with LANA was highlighted by dot box. Percentage of co-localization from 10 counted cells.

To further confirm that LANA^SIM^ is required for LANA to interact with SUMO-2, we investigated the subcellular localization of LANA and SUMO-2 by using the wild type or SIM-deleted NC truncated mutant of LANA. Consistently, the results showed that SUMO-2 localizes predominantly to the outside compartment of chromosomal DNA when expressed alone (Figure 2A). However, we observed 64.8% co-localization of SUMO-2 with LANA on chromosomal DNA in the presence of wild type LANA (Figure 2B, upper panels). In contrast, only 0.5% SUMO-2 was co-localized with LANA on chromosomal DNA when the SIM motif of LANA was deleted (ΔSIM) (Figure 2B, lower panels). To further demonstrate this point, the sequential ChIP assays were performed in the presence of TR DNA. The results also showed that wild type not SIM-deleted mutant dramatically associated with SUMO-2 on DNA compartment (Figure 2C). This suggests that LANA^SIM^ is required for LANA to co-localize with SUMO-2 on chromosomal DNA. In consistence, the results from immunofluorescence analysis of endogenous LANA and SUMO-2 in PEL cells further supported the notion that hypoxia reduces co-localization of LANA with SUMO-2 (Figure 2D).

### Poly-SUMO2 modified KAP1 is a key component of the LANA^SIM^-mediated regulatory complex

**Figure 3:**
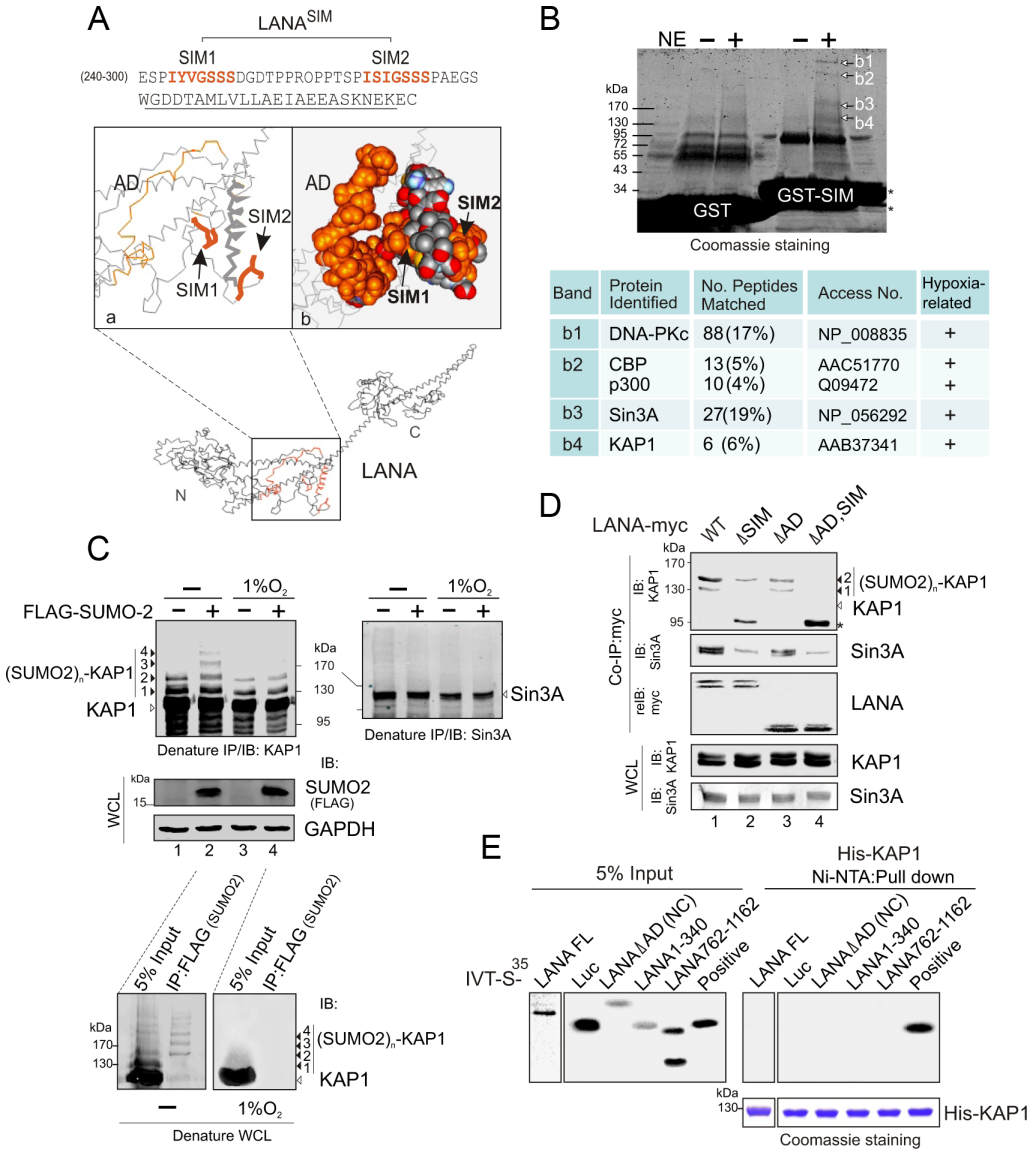
The LANA^SIM^ motif interacts with hypoxia-sensitive poly-SUMO2 modified chain of KAP1. (**A**) Illustration of the position of LANA^SIM^ and LANA^AD^ in the 3D structure of LANA predicted by Robetta server (http://robetta.bakerlab.org/). The two SIM motifs (amino acids 240 to 300) and acidic domain (AD, aa 595 to 699) are marked in orange. (**B**) Identification of proteins with SUMO-2 modification that specifically associate with LANA^SIM^. *Top panel*, Coomassie staining image of bands b1, b2, b3 and b4 from the gradient gel; *Bottom panel*, Protein identification of bands b1, b2, b3 and b4 by MALDI-TOF-MS (matrix-assisted laser desorption ionization time-of-flight mass spectrometry). Accession # in NCBI protein Database; sequence coverage from MALDI-TOF data; and the relevance of each protein with both SUMO modification and hypoxic response is indicated. (**C**) poly-SUMO2 modification of KAP1 is diminished by hypoxic stress. HEK293 cells transfected with plasmids expressing FLAG-SUMO-2 or vector alone was individually treated with or without hypoxia (1% O_2_) at 24 hr post-transfection for overnight before harvest. Whole cell lysates (WCL) were subjected to directly immunoblotting (IB) with FLAG or GAPDH antibody, or denature immunoprecipitation (IP) followed by immunoblotting with KAP1 or Sin3A antibody as indicated. Solid arrows and lines indicate the poly-SUMO2 modified KAP1. Denature immunoprecipitation with FLAG antibodies was used as positive control to show (SUMO2)n-KAP1 (n = 1, 2, 3, or 4 copies) at the bottom panel. (**D**) Deletion of LANA^SIM^ disrupts the association of LANA with poly-SUMO2 modified KAP1 and native Sin3A. HEK293 cells were co-transfected with plasmids expressing wild type (WT) LANA or its mutants (ΔSIM, ΔAD and ΔAD,SIM) with myc tag in the presence of FLAG-SUMO-2. At 48 hr post-transfection, cell extracts were subjected to directly immunoblotting (IB) or immunoprecipitation (IP) with anti-myc antibody followed by immunoblotting against KAP1 or Sin3A. The asterisk denotes the degraded KAP1. (**E**) KAP1 does not directly interact with LANA *in vitro*. *In vitro*-translated, radiolabeled full length (FL) LANA or its different truncated mutants was individually incubated with His-KAP1 recombinant protein with Nickel-Agarose beads. Bound complexes were analyzed by autoradiography. The Coomassie staining of His-fused KAP1 recombinant protein is shown at the bottom. Luciferase (Luc) was used as negative control and FLAG-Mdm2 as positive control of KAP1 binding protein. Input was used at 5%.

To identify which proteins with SUMO-2 modification interact with the LANA^SIM^ motif, we first analyzed the 3D structure of LANA by Robetta prediction software (http://robetta.bakerlab.org/). Strikingly, we found that the region which contains residues 240 to 300 forms a separate domain with two sub-SIM (SIM1 and SIM2) motifs individually distributed on each side (Figure 3A). This potentially provides a “riding-horseback” like platform important for LANA interaction with poly-SUMO-2. In addition, we also noticed that the AD domain of the central region is located very close to the LANA^SIM^ motif (Figure 3A), which may explain why the deletion of the AD domain decreases the interaction of LANA^SIM^ with poly-SUMO-2. Based on this predicted 3D structure of the SIM domain, we generated a GST-SIM fusion protein by using the residues 240 to 300 region and performed GST pulled down assay with nuclear extracts from 293 cells expressing exogenous SUMO-2. The results from gel fractionation results showed that at least four unique bands (b1, b2, b3 and b4) were pulled down by the GST-SIM protein (Figure 3B, upper panel). Analysis of these bands by mass spectrometry identified five proteins: DNA-PKc, the transcriptional coactivators CBP and p300, and the corepressors Sin3A and KAP1, respectively (Figure 3B, lower panel). Interestingly, previous studies have suggested that these five proteins are responsive to hypoxic stress [34], [35], [36], [37]. In addition, except for DNA-PKc, the other four transcription factors were reported to undergo SUMO modification [38], [39], [40], [41]. To elucidate which proteins in this complex are critical for interaction with the LANA^SIM^ motif through poly-SUMO-2, we first performed similar GST pull-down assays by using GST fusion with the wild type (WT) or the SIM-deleted (ΔSIM) mutant of LANA N terminus 1–329, following western blot with specific antibodies for each of the five proteins. The results showed that deletion of the LANA^SIM^ motif results in a dramatically dissociation of KAP1 and Sin3A with the LANA^SIM^-mediated complex (data not shown). To further verify if KAP1, Sin3A or both undergo SUMO-2 modification as LANA^SIM^ targets are impaired by hypoxia stress, HEK293 and BJAB cells individually transfected with FLAG-SUMO-2 or vector were treated with or without hypoxia and subjected to denature IP of KAP1 and Sin3A. The results showed that KAP1 but not Sin3A was dramatically modified by SUMO-2 (1, 2, 3 and 4 copies of SUMO-2 according to 19 kDa molecular weight of SUMO-2) in the presence of FLAG-SUMO-2. Furthermore, this modification was blocked by hypoxia (Figure 3C, compare lanes 2 with 4, similar results were observed in BJAB cells). Intriguingly, although the denatured form of SUMOylated KAP1 (de-suKAP1) was not pulled down by the SIM domain of LANA fusion with GST in vitro (data not shown), the native co-immunoprecipitation assays showed that the LANA^SIM^ motif within full length LANA or its NC truncated mutant had a higher affinity with 2×SUMO-2 modified KAP1 than 1×SUMO-2 modified one (Figure 3D, lanes 1 and 3), and deletion of the LANA^SIM^ motif dramatically reduces or completely abrogates the interaction of LANA with 1 or 2× SUMO-2 modified KAP1 (Figure 3D, compare lanes 1 and 2 with 3 and 4). Unexpectedly, deletion of SIM motif in both full length LANA and its NC truncated mutant led to appearance of a degraded band (∼95 kDa) (Figure 3D, lanes 2 and 4 indicated by asterisk). The result of less association of LANA with native Sin3A once the SIM motif was deleted (Figure 3D, middle panel, lanes 2 and 4), further supports the notion that the poly-SUMO2 modified chain of KAP1 is the critical one targeted by the LANA^SIM^ motif. In addition, the results of in vitro binding assays showed no direct interaction of His-KAP1 with *in vitro* translated proteins of full length LANA and its different truncated mutants (Figure 3E), suggesting that LANA predominantly interacts with poly-SUMO2 modified KAP1 through the LANA^SIM^ motif.

**Figure 4:**
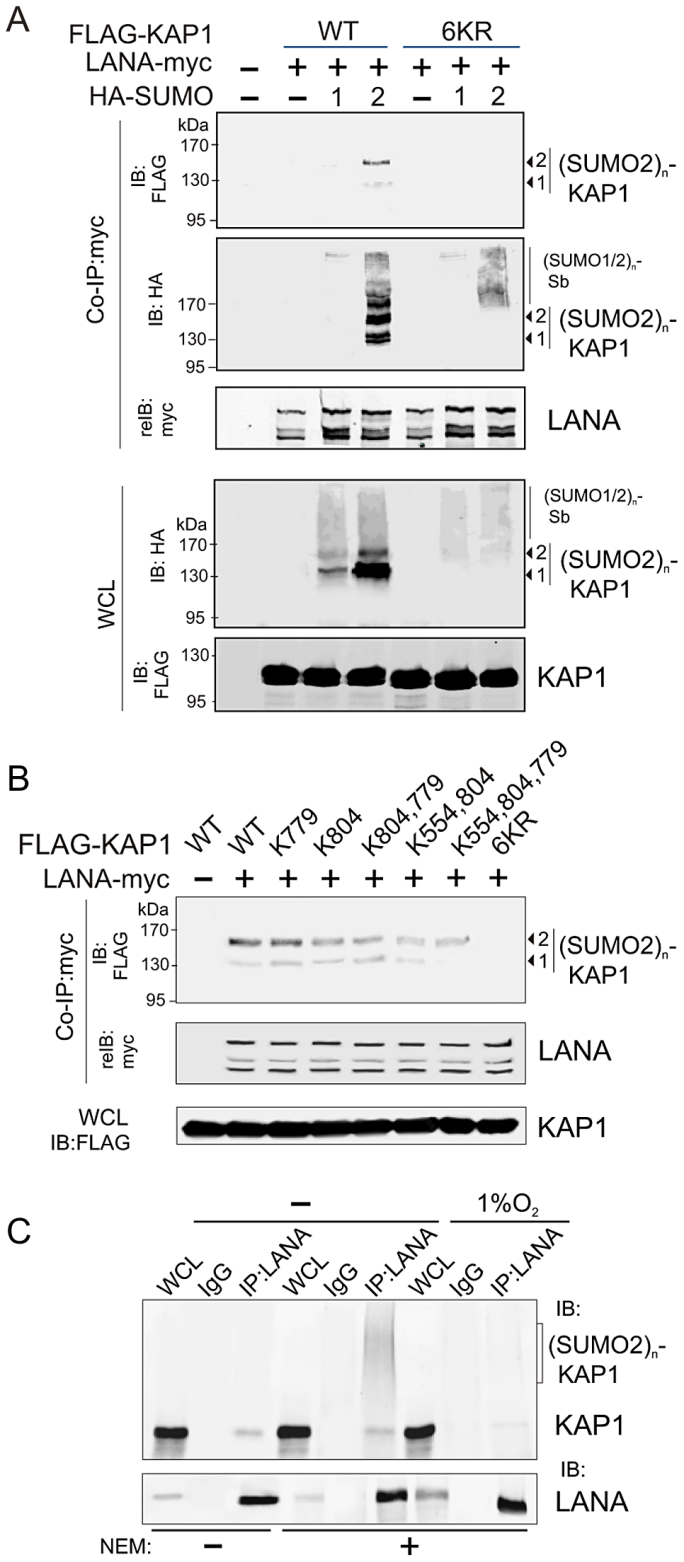
LANA recognizes the poly-SUMO2 modified chain on Lysine 779 and Lysine 804 of KAP1. (**A**) LANA interacts with poly-SUMO2 not poly-SUMO1 modified chain of KAP1. HEK293 cells were co-transfected with expression plasmids as indicated in the figure. At 24 hr post-transfection, whole cell lysates (WCL) were subjected to directly immunoblotting (IB) or co-immunoprecipitated (co-IP) followed by immunoblotting as indicated. (**B**) LANA is able to individually bind with poly-SUMO2 modified chain on lysine 779 and lysine 804 of KAP1. HEK293 cells were co-transfected with expression plasmids as indicated in the presence of HA-SUMO-2, and subjected to similarly analyze as shown in *panel A*. (C) Endogenous KAP1 associates with LANA in PEL cells and responds to hypoxic stress. Whole cell lysate of BC3 cells treated with or without hypoxia (1% O_2_) for overnight, were individually subjected to immunoprecipitation with normal IgG or LANA antibodies followed by immunoblotting as indicated in the figure. *N*-ethylmaleimide (NEM, deSUMOylation inhibitor) was present or absent in cell lysates.

To further confirm that LANA does bind with the SUMO-2 modified KAP1, the wild type KAP1 and its mutant 6KR (all 6 putative SUMOylated lysines are mutated to arginine) with FLAG tag were individually used to perform co-IP with myc-tagged LANA in the presence of HA-SUMO-1, HA-SUMO-2 or vector alone. The results showed that as observed with endogenous KAP1 and SUMOylation, LANA strongly associated with 1 or 2×SUMO-2 modified KAP1 with greater affinity for the 2×SUMO2-KAP1, although there was more 1× SUMO-2 modified KAP1 seen in the whole cell lysate (Figure 4A). Furthermore, the results of LANA association with 1 or 2×SUMO-2 modified KAP1 with the lysines mutated indicated that LANA^SIM^ binds preferentially with poly-SUMO2 modified KAP1 and that these lysines contributed to KAP1 SUMOylation to various degrees with a complete loss of association when all 6 lysines were mutated (Figure 4B). In PEL cells with hypoxia, less association of SUMO2-modified and native KAP1 with endogenous LANA than that in normoxia (Figure 4C), further demonstrated that the association of LANA with KAP1 is sensitive to hypoxic stress.

### The LANA^SIM^ motif is important for SUMO-2 modification of LANA

To determine whether the LANA^SIM^ motif of LANA contributes to its SUMO modification, we performed native and denatured (which removes non-covalent binding but retains covalent binding of SUMO modification), co-IP assays by using full length LANA or its LANA^SIM^-deleted mutant (LANA^ΔSIM^) coexpressed with exogenous SUMO-1 or SUMO-2. Ubc9 was used as a positive control in this assay. The results showed that LANA can be modified by both SUMO-1 and SUMO-2 (supplementary Figure S2, upper panels). Importantly, the signals indicating that LANA was SUMO-2 modified were greater than that seen for SUMO-1 (supplementary Figure S2, upper panel, lanes 5 and 6). Consistent with our findings that the LANA^SIM^ motif associates with SUMO-2 but not SUMO-1, and the LANA^SIM^ motif deletion resulted in a remarkable reduction of SUMO-2 modification of LANA (supplementary Figure S2, compare lanes 6 with 8). In contrast, the levels of SUMO1-modified LANA were relatively unchanged in the ΔSIM mutant compared to WT LANA (supplementary Figure S2, compare lanes 5 with 7).

As the LANA^SIM^-associated poly-SUMO2 modified KAP1 is highly sensitive to hypoxic stress, we wanted to know if SUMO-2 modification of LANA is also impaired by hypoxia. We monitored the SUMO-2 modified levels of endogenous LANA by *in vivo* SUMOylation assays in PEL cells under normoxic and hypoxic conditions. The KSHV negative (no LANA) BJAB cell line was used as a negative control. The results showed that the levels of both SUMO-2 modified LANA (suLANA) and native LANA associated with SUMO-2 in BC3 cells are also dramatically reduced under hypoxic conditions (supplementary Figure S3). This corroborated the above data that the LANA^SIM^ motif does contribute to SUMO-2 modification of LANA due to dissociation from hypoxia-sensitive SUMOylated KAP1.

To determine which residues of LANA targeted for SUMOylation, we tested the SUMOylated pattern of different amino or carboxyl truncated forms of LANA (to avoid the problem of resolution of the SUMO-modified bands linked to full length LANA) in the presence of SUMO-1 or SUMO-2 by *in vivo* SUMOylation assays. The results showed that when coexpressed with SUMO-1 or SUMO-2, only the central region deleted (NC) and carboxyl terminus mutants of LANA but not its amino terminus mutant appear to have SUMO-1 or SUMO-2 modified forms of LANA (with slower migration compared to unmodified LANA) (data not shown). To identify which specific residues are SUMO modified, we analyzed the carboxyl terminal sequence of LANA according to the SUMOylated consensus sequence ΨKxE/D [27], and identified two lysine residues 1081 and 1140 which are most potentially targeted for SUMOylation (supplementary Figure S4, right panel). Follow-up SUMOylation assays in cells indicated that lysine 1140 of LANA is the key residue for both SUMO-1 and SUMO-2 modification (supplementary Figure S4, left panel).

In view of the fact that the LANA^SIM^ motif contributes to SUMOylation of LANA, to further verify if the LANA^SIM^-associated complex (including KAP1) acts as a bridge for the amino terminus of LANA to interact with its carboxyl terminus, and in turn enhances SUMO modification of carboxyl terminus of LANA, we performed co-IP assays by expressing FLAG-tagged C terminus of LANA (LANA-C) with myc-tagged amino terminus (WT or ΔSIM) of LANA (LANA-N) in 293 cells. The results showed that the association of the amino terminus with the carboxyl terminus of LANA is dependent on the presence of SIM motif to a large extent (supplementary Figure S5A, lanes 2 and 3). In addition, co-expression of the amino with the carboxyl terminus of LANA dramatically enhances the SUMOylation at its carboxyl terminus (supplementary Figure S5B, lanes 1 and 2). Furthermore, the levels of the amino terminus-induced SUMOylation of the carboxyl terminus were significantly decreased when LANA^SIM^-associated KAP1 was knocked down (supplementary Figure S5B, compare lanes 2 with 4). This further supports the notion that the LANA^SIM^ motif and KAP1 play a role in SUMOylation of LANA.

### The LANA^SIM^ motif is essential for KSHV episome maintenance, silencing RTA expression and blocking lytic replication

**Figure 5:**
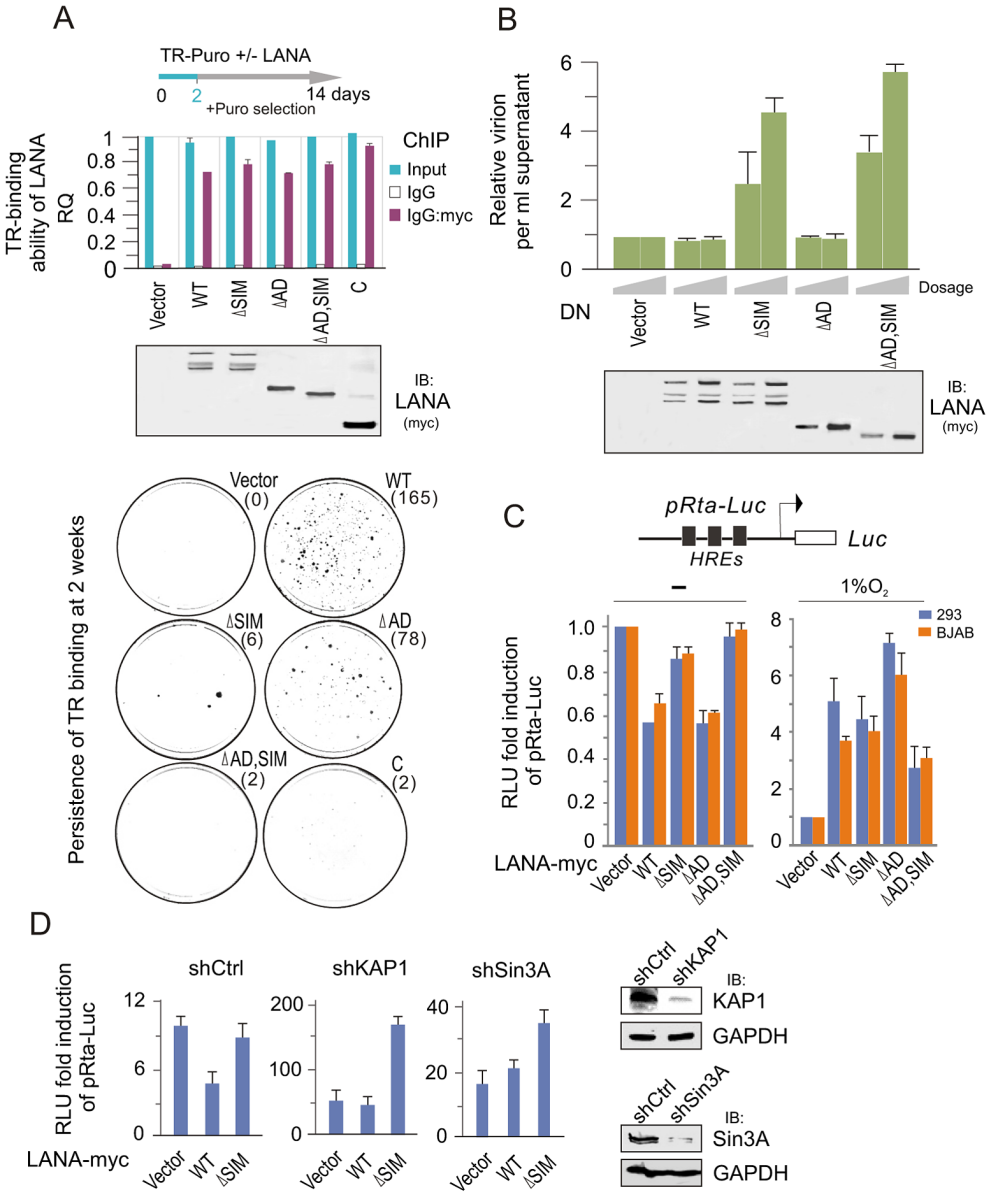
The LANA^SIM^ motif is important for maintenance of KSHV episome. (**A**) Deletion of LANA^SIM^ does not affect the TR-binding ability of LANA but lose the capacity of LANA-mediated TR maintenance. HEK293 cells were co-transfected TR-puromycin plasmid with wild type or different mutants of myc-tagged LANA or vector alone as indicated. The equal transfection efficiency is monitored by GFP co-expression. At 48 hr post-transfection, cells were subjected to ChIP assays with normal mouse IgG or mouse anti-myc(9E10) antibodies followed by real-time quantitative PCR with TR primer (*upper panel*). The level of TR per transfection was compared to vector alone and shown as input. The results of TR-binding ability of LANA were presented after normalization with the protein levels of LANA. For colony formation assays of LANA-mediated TR maintenance, two thousands transfected cells at 2 day post transfection were seed and selected with 2 µg/ml puromycin for 14 days (*lower panel*). The average of colonies quantified from duplicate experiments is shown in the figure. The expression of wild type LANA and its mutants are presented by western blot assay at the middle panel. (**B**) Dominant negative (DN) mutant of LANA with SIM deletion reactivates KSHV lytic replication. HEK293/Bac36 stable cells were transfected with different dosage (10, 20 µg) of expressing plasmids as indicated in the figure. At 48 hr post-transfection, the relative virion per ml supernatant of transfected cells was detected by quantitative PCR with TR as target. GAPDH was used as internal control. The expression of wild type LANA and its mutants are presented by western blot assay at the bottom panel. (**C**) Reporter assays of the Rta promoter in the presence of LANA under normoxic and hypoxic conditions. HEK293 and BJAB cells were individually co-transfected HRE-containing Rta promoter driven Luciferase reporter (pRta-Luc) with wild type (WT) LANA-myc or its mutants (ΔSIM, ΔAD, or ΔAD,SIM). At 24 hr post-transfection, cells were exposed with or without 1% oxygen for overnight before harvest. The results were presented by the RLU (relative luciferase unit) fold compared to pRta-Luc with vector alone. Data is presented as means±SD of three independent experiments. (**D**) Knockdown of KAP1 or Sin3A blocks LANA^SIM^-mediated inhibition of LANA on Rta promoter. HEK293 cells with constitutive knockdown of KAP1 (shKAP1), Sin3A (shSin3A) or a scrambled negative control (shCtrl) were co-transfected pRta-Luc with LANA-myc (WT or ΔSIM) or vector alone. The pRta-Luc parental vector (pGL2-basic) alone was used as a control. Cells were collected at 48 hr post-transfection and analyzed by reporter assays as described in panel C.

Since LANA plays a critical role on maintenance of viral episome during latency, we wanted to know if the LANA^SIM^ deletion impairs the ability of LANA binding with the TR and the persistence of the viral episome during cell passage. To this end, we performed chromatin immunoprecipitation assays by using wild type (WT) LANA or a series of its mutants (ΔSIM, ΔAD, ΔAD, SIM and C) co-expressed with the TR-Puromycin plasmid in 293 cells for 48 hours. The results showed that there were no significant difference between wild type LANA and other mutants (Figure 5A, upper panels). To further determine if the persistence of LANA-binding TR was affected, the transfected cells of LANA and TR-Puromycin were subsequently subjected to colony formation assay where cells with TR plasmid were selected with Puromycin for 2 weeks (a greater number of colonies would indicate higher efficiency of the TR persistence). The results showed that both wild type (WT) LANA and its NC truncated mutant (ΔAD) presented the highest efficiency of TR maintenance, while both LANA^SIM^-deleted mutants ΔSIM and ΔAD, SIM led to a dramatic loss of TR colonies (Figure 5A, lower panels). In contrast, due to the absence of the amino terminus of LANA for tethering to host chromosomal DNA [42], there was little or no TR colony formation seen in the carboxyl terminal truncated mutants (C) which were similar to vector alone (Figure 5A, lower panel). These results indicate that the LANA^SIM^ motif is an important contributor to LANA's role in maintenance of the KSHV episome.

To answer if the LANA^SIM^ motif also contributes to inhibition of lytic replication, we transiently transfected LANA^SIM^ -deficient mutants of LANA as a dominant negative into 293 cells stably carrying the complete KSHV-Bac36 (viral genome), and then monitored for intracellular viral episomal DNA by quantitative PCR analysis. Results with wild type LANA, NC truncated mutant ΔAD or vector alone as control dramatically reduced virion DNA production. However, we also showed that both the LANA^SIM^-deleted mutants ΔSIM and ΔAD, SIM dramatically enhanced viral episome replication (Figure 5B). This strongly suggested that in addition to maintenance of the viral episome, the LANA^SIM^-associated complex including KAP1 and Sin3A are also important for blocking lytic replication. To determine whether this LANA^SIM^ -dependent inhibition of lytic replication is due to silencing RTA (the key activator of the KSHV lytic life cycle) expression, the RTA promoter was subjected to luciferase reporter assays in the presence of wild type LANA, its mutants with or without LANA^SIM^ deletion (ΔSIM, ΔAD, and ΔAD,SIM) or vector alone. The results showed that the LANA^SIM^-deletion alone (ΔSIM) recovered about 60% of the inhibition of wild type LANA whereas the ΔAD mutants had negligible effect (Figure 5C, left panel). However, the ΔAD,SIM mutant led to an almost complete reversal of the inhibitory activities (Figure 5C, left panel). In contrast, hypoxic stress efficiently reversed the inhibitory function of LANA resulting in transactivation of the RTA promoter regardless of whether the wild type LANA or the NC truncated mutants with or without LANA^SIM^ deletion (ΔAD, ΔAD,SIM) were used in the assay (Figure 5C, right panel). This further supports the notion that the LANA^SIM^-mediated complex (including KAP1) is involved in the inhibitory function of LANA in RTA expression and is sensitive to hypoxia. To further confirm that LANA^SIM^-mediated KAP1 and Sin3A are indeed required for LANA to repress the RTA promoter through the LANA^SIM^ motif, we performed RTA promoter reporter assays in the presence or absence of LANA in both BJAB and 293 cell lines with Lentivirus-mediated constitutive knockdown of KAP1 or Sin3A or nonspecific control. Consistent with our findings, the results showed that knockdown of KAP1 or Sin3A efficiently blocked the inhibitory function of LANA on the RTA promoter in normoxia (Figure 5D). This is consistent with our observation that the dominant negative LANA^SIM^-deleted mutant does in fact enhance KSHV virion production (Figure 5B).

**Figure 6:**
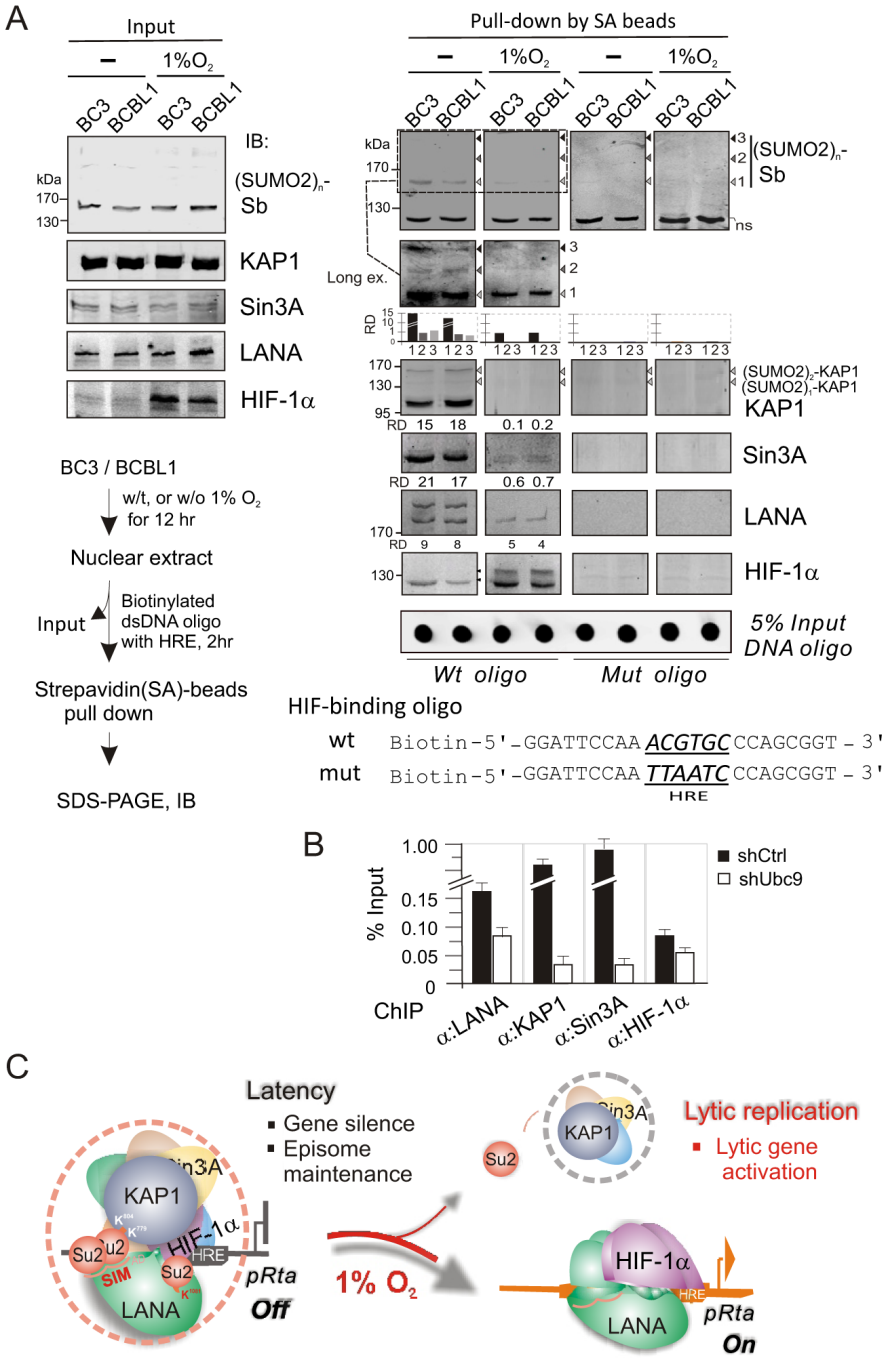
The LANA^SIM^-mediated complex with KAP1 and Sin3A is critical for KSHV latency maintenance and response to hypoxia. (**A**) *In vitro* DNA-binding assays of HIF-1α in complex with the (SUMO2)n-KAP1, Sin3A, and LANA under normoxic and hypoxic conditions. Nuclear cell extract from BC3 or BCBL1 with or without 1% oxygen treatment for overnight, was individually incubated with wild type or mutated HIF-1α-binding DNA oligo, and the input and precipitations were immunoblotted (IB) with antibodies as indicated, respectively. The brief protocol is shown in *Top right panel*. The relative density (RD) of native KAP1, Sin3A, LANA and SUMO-2 binding and 5% Input of DNA oligo are presented. (**B**) The ChIP assay of LANA, KAP1, Sin3A and HIF-1α by using the HRE-containing region of ORF50 promoter with or without Ubc9 knocked down. Chromatin DNAs were prepared from KSHV-positive cell line BC3 with Ubc9 (shUbc9) and scramble (shCtrl) knockdown before harvest. (**C**) Schematic presents the role of LANA^SIM^-mediated transcriptional inhibitory complex including KAP1 and Sin3A in RBP-Jκ and HIF-1α-binding sites for KSHV gene silence and episome maintenance during latency. In hypoxic stress, the loss of SUMOylation status (particularly poly-SUMO2-KAP1) results in the dissociation of KAP1 and Sin3A from the RBP-Jκ-LANA^SIM^-HIF-1α complex; this in turn dramatically increases RTA expression and globally activates KSHV lytic replication and produces virion progeny.

To determine whether the hypoxia-induced functional switch of LANA is due to the dissociation of the LANA^SIM^-associated proteins (like KAP1 and Sin3A) from the regulatory complex LANA-HIF-1α particularly on the HIF-1α-DNA binding sites, we performed a biotin-labeled DNA oligo pull-down assay *in vitro* by incubating the nuclear extract with wild type or a mutant of the HIF-binding site-(mut) HRE DNA oligo. The specific proteins bound to HRE were pulled-down by Strepavidin agarose beads and were subjected to western blot analysis. The results showed that in normoxia the LANA^SIM^-associated proteins KAP1 and Sin3A in the regulatory complex of LANA and HIF-1α bound to HRE (Figure 6A). However, in hypoxia, KAP1 and Sin3A as well as SUMO-2 (where the SUMO-2 position corresponds to the pattern of poly-SUMO2 modified KAP1 as calculated by molecular weight) were dramatically released from the LANA-HIF-1α/HRE complex (Figure 6A, right panels). Notably, we observed that a modified form (potential SUMO-2 modification based on the pattern of SUMO2 modified LANA) of LANA was also dissociated from the LANA-HIF-1α/HRE complex while interestingly HIF-1α appears with an additional modified slower migrating band (Figure 6A, right panels). To further determine if SUMOylation is important for DNA binding of the LANA^SIM^-mediated complex, the ChIP assays of HRE region within the ORF50 promoter were carried out in BC3 cells with or without transiently Ubc9 knockdown. The results showed that inhibition of Ubc9-mediated SUMOylation dramatically reduced both KAP1 and Sin3A association with LANA-HIF-1α complex at the HRE DNA (Figure 6B). Taken together, this data above strongly support our hypothesis that KAP1 and Sin3A particularly SUMO-2 modified forms are indeed sensitive to hypoxic stress and are recruited by the LANA^SIM^ motif during KSHV latent infection to silence the RTA promoter under normoxic conditions.

## Discussion

Despite the fact that latent infection is the dominant form of KSHV infection which leads to transformation of the host cell, reactivation from latency is also important for the growth and dissemination of viral associated tumor cells [13]. In addition to chemical triggers like phorbol esters and sodium butyrate, reactivation from KSHV latency can also be initiated by various physiological and environmental factors including hypoxia [43]. These agents are thought to directly or indirectly target the transcriptional activation of the promoter which drives the KSHV RTA (ORF50) immediate-early gene. Our previous studies showed that LANA encoded by KSHV cooperates with HIF-1α bound to the HREs of the RTA promoter to induce lytic replication under hypoxic conditions [18]. Interestingly, we also found that LANA stabilizes HIF-1α in normoxia by blocking the negative regulators VHL and p53 and inducing nuclear accumulation [20], [44]. To investigate how LANA switches from its inhibitory function to transcription activation at the RTA promoter, we now report that LANA has a unique SUMO2-interacting motif (LANA^SIM^) and that this LANA^SIM^ motif specifically recruits a transcriptional inhibitory complex containing KAP1 and Sin3A for maintenance of the KSHV episome, as well as silencing of lytic gene (i.e. RTA) expression under normoxic condition. Of this complex, the poly-SUMO2 modified KAP1 is a critical factor targeted by the LANA^SIM^ motif. In hypoxia (i.e. 1% O_2_ in this study), loss of SUMOylation of KAP1 leads to the LANA^SIM^-associated complex particularly KAP1 and Sin3A dissociation from the LANA-HIF-1α/HRE complex, as well as deSUMOylation of LANA itself, which open the active form of LANA-HIF-1α, and in turn induces lytic gene expression and reactivates lytic replication in response to hypoxic stress (Figure 6C). Interestingly, despite the fact that much dramatically dissociation of KAP1 and Sin3A from HIF-1α/HRE complex in the less SUMOylation status upon hypoxic stress, we did also observe that less native LANA association with HIF-1α/HRE complex to some extent in hypoxia when compared with that in normoxia, which could be due to the less stability of LANA without SUMO modification in hypoxia.

The modification of proteins by SUMO conjugation has been demonstrated as a reversible process important for regulating the function of many target proteins [45], [46]. The consequences of SUMO modification are not completely understood but have been shown to control various facets of protein function, including gene transcription, cell cycle, DNA repairs and protein localization [45], [46]. The most recent discovery of SUMO-interacting motif leads further interpretation to the roles that SUMO modification can play on protein-protein interaction, conformational changes and subcellular localization [31], [32], [33]. In this study, we discovered that LANA has a unique SUMO-2 interacting motif (LANA^SIM^) containing two SIM motifs with partial homology to cellular SIM motifs. Furthermore, this LANA^SIM^ motif specifically associated with poly-SUMO-2 instead of poly-SUMO-1. The finding of poly-SUMO2 (2×SUMO-2) modified KAP1 associated with the LANA^SIM^ motif indicates that this interaction requires the intact 3D structure of the LANA^SIM^ motif. Notably, since SUMO-2 signaling is only activated due to extracellular stress, LANA may possess a unique SUMO-2 instead of SUMO-1 interacting motif to usurp the host SUMOylation pathway by mimicking stress signaling (This could be the reason why LANA^SIM^ greatly associates with certain target proteins instead of others with SUMO-2 modification), thus creating a cellular environment favorable for viral infection. Other instances including cytomegalovirus IE2 [47], human papillomavirus E2 [48], Epstein-Barr virus BZLF1 [49], and KSHV K-bZIP [50], [51], further support this notion. Our findings which showed that the AD domain of LANA's central region partially contributes to the LANA^SIM^-associated protein-protein interaction, which subsequently enhances SUMO modification of LANA, supports previous discoveries that acidic residues located downstream from the core SUMO modification sites will enhance the efficiency of SUMOylation of target proteins [52]. In addition, we did not find a similar LANA^SIM^-motif sequence within EBNA1 (the LANA homologous antigen encoded by Epstein-Barr virus), or ORF73 encoded by HVS C488, A11 or MHV68. In contrast, distinct from recent report indicating that a SIM motif is located at carboxyl terminus of LANA and required for SUMOylation of histone H2B [53], the hypoxia-based LANA^SIM^ discovered at amino terminus of LANA in our study could mainly target high-molecular-weight proteins (i.e. KAP1) instead of low-molecular weight ones (i.e. H2B). The fact that the LANA^SIM^ motif is required for interaction between amino and carboxyl terminal domains of LANA, further suggests that the LANA^SIM^ motif could serve as an adaptor for both intra-molecular and inter-molecular interactions of LANA as well as with other SUMO target proteins.

The LANA^SIM^ motif recruits a specific transcriptional regulatory complex which includes corepressors, coactivators, and DNA binding proteins. KAP1 (also known as TIF1β or TRIM28) was identified as a key component of the complex. It is well known that KAP1 is a universal corepressor protein from the Kruppel associated box (KRAB)-domain containing zinc finger proteins, the largest family of transcriptional silencers in the human genome [54], [55]. KAP1 itself cannot bind DNA directly and recruits or coordinates the assembly of several chromatin-remodeling proteins including the histone deacetylase complex (NCoR1) [56], and the heterochromatin protein 1 (HP1) family member [57]. Emerging evidence suggests that post-translational modifications, such as SUMOylation and phosphorylation can affect the ability of KAP1 to condense or relax chromatin [41], [58]. SUMOylation at Lys^554^, Lys^779^ and Lys^804^ of KAP1 generates a binding platform for SETDB and HADC1 to condense chromatin [58] (This may explain why there was a lower percentage of KAP1 matched peptide than other proteins like Sin3A in MALDI-TOF-MS assay). In response to hypoxia, KAP1 was previously shown to be phosphorylated in an ATM-dependent manner on Ser^824^, and phosphorylated KAP1 spreads rapidly throughout the chromatin [59]. In this study, the result of less SUMOylation of KAP1 in hypoxia provides further evidence that SUMOylation of KAP1 is hypoxia-sensitive and is linked to chromosome condensation and gene silencing. Therefore, knocking down KAP1 will lead to constitutive relaxation of cellular and viral chromatin for gene transcription and expression [60]. Taken together, these results provide a better understanding of the mechanism by which hypoxia drives the dissociation of KAP1 from LANA containing complexes for transactivation of target gene promoters.

Another key transcriptional co-repressor identified associated with the LANA^SIM^ motif was Sin3A. Like KAP1, Sin3A also functions as a co-repressor by recruiting HDAC1 for gene silencing [61]. Although Sin3A is shown as a substrate of TOPRS SUMO E3 ligase [40], it was difficult to detect the SUMOylated form of Sin3A directly associated with the LANA^SIM^ motif. However, the evidence of Sin3A knockdown results in dramatic attenuation of the enhancement of the transcriptional activity at the RTA promoter, indicating that Sin3A is also a key component in the LANA^SIM^-associated complex. In addition, both transcriptional co-activators p300 and CREB-binding protein (CBP) were also pulled down by the LANA^SIM^ domain. However, albeit that the LANA^SIM^ deletion did not significantly impair their association with LANA, which minimizes a critical role for these co-activators in the LANA^SIM^-associated silencing complex, their contributions cannot be completely ruled out. The aim of LANA targeting p300 and CBP is likely to take advantage of the amount of p300 and CBP in order to compete with other DNA bound transcriptional factors and so is important for balancing the repression and activation at these major lytic promoters.

Although a recent report showed that the lytic protein vPK blocks the SUMOylation of KAP1[62], the fact that deSUMOylation of KAP1 in hypoxia not only occurs in KSHV positive cells but also negative cells, argues against the possibility that hypoxia may up-regulate other viral antigens which in turn lead to the loss of SUMOylation of KAP1. In addition, the evidence that both KAP1 and Sin3A is released from the LANA-HIFα complex binding to the HRE elements during hypoxic stress provides additional questions for further investigation: 1) Is the deSUMOylation enzyme SENP up-regulated by hypoxia stress, and does deSUMOylation of KAP1/Sin3A by SENP lead to loss of KAP1/Sin3A binding with LANA in the silencing complex; 2) Does native or modified HIF-1α bind to LANA and block the access of KAP1 and Sin3A to the LANA^SIM^ motif?

In summary, our study provides new mechanistic insights into the programmed regulation of the KSHV genome during latent and lytic replication controlled by the major latent antigen LANA for gene silencing or activation based on a specific LANA^SIM^-associated complex. Particularly, the discovery that the LANA^SIM^-associated complex is highly sensitive to hypoxia (a physiologically relevant environmental condition) and so provides a potential therapeutic target against KSHV-associated cancers.

## Materials and Methods

### DNA constructs and antibodies

Plasmids encoding full length LANA with myc tag and its truncation mutants NC (1–329∧925–1162), N1 (1–435), N2 (1–233), and N3 (1–340) were previously described [20]. GFP-C1(930–1162)-myc and C(842–1162)-FLAG of LANA were obtained by ligating *Kpn*I/*Eco*RV PCR fragments into pA3M-GFP and pA3F vector, respectively. GST-SIM(240–300), GST-LN_1–329_,and GST-LN_1–329_ΔSIM were generated by ligating *Kpn*I/*Eco*RI PCR fragments into the derivative of pGEX-2TK (Introduced a *Kpn*I restriction enzyme site in open reading frame of GST). HA-Ubc9 was generated by ligating *Bam*HI/*Eco*RI PCR fragments into the plasmid of pcDNA3-HA. Plasmids pRta-Luc, HA-SUMO-1/2, FLAG-SUMO-1/2, GFP-SUMO-1/2 were described as previously [18], [22]. His-SUMO-1 and His-SUMO-2 were individually generated by ligating *Bam*HI/*Hin*dIII and *Bam*HI/*Eco*RI PCR fragments into the plasmid of pET-15b. His-KAP1 was generated by *Nde*I/*Bgl*II PCR fragment into pET-28a vector with *Nde*I/*Bam*HI digestion. Briefly, mutants of full length LANA-myc (ΔSIM), NC-myc (ΔSIM), LANA-N3-myc (ΔSIM1, ΔSIM2, ΔSIM, 3IA and 3SA), GFP-LANA-C1-myc (K1081R), and -(K1140R), FLAG-KAP1 with Lysine 779 (K779), Lysine 804 (K804), Lysine 779,804 (K779,804), Lysine 554,804 (K554,804) or Lysine 554, 804,779 (K554,804,779)only were generated by PCR site-directed mutagenesis. All constructs were confirmed by direct DNA sequencing. Wild type FLAG-KAP1 and its 6KR mutant were kindly provided by Dr. Frank J Rauscher III from the Wistar Institute, Philadelphia [58].The SUMO-1 (Y299), DNA-PKcs (18-2), KAP1 (20C1) and β-actin (#8226) antibodies were from Abcam. HIF-1α antibodies were from BD transduction laboratory. Sin3A (AK-11), SUMO-2/3 (FL-103), GFP (F56-BA1), p300(C-20), and CBP(A-22) were purchased from Santa Cruz Biotech. Inc. GAPDH (G8140-01) antibodies were from US Biological Inc.. Mouse monoclonal antibodies against LANA (LANA1, or LNA1 rat mAb from Advanced Biotech Inc.), hemagglutinin (12CA5), FLAG (M2), and myc (9E10) were used as described previously [20].

### Cell culture, transfection and hypoxic incubation

KSHV-negative (BJAB) and positive (BC3, BCBL1) B lymphoma cells were cultured in RPMI supplement with 7% FBS (Hyclone). HEK293 cells and 293/Bac36 stable cell line (established by 293 cells transfected with wild type KSHV BACmid [Bac36] and selected by 150 ng/ml Hygromycin B [Sigma]) were maintained in Dulbecco's modified Eagle's medium (DMEM) containing 5% FBS (Hyclone). Transfections were performed by electroporation with a Bio-Rad Gene Pulser in 0.4 cm-gap cuvettes at 220 Volts and 975 microfarads. Cells were grown in a humidified atmosphere at 37°C at gas tensions of 21% O_2_/5% CO_2_ for normoxic incubation and 1% O_2_ (or 21% O_2_ with 100 µM CoCl_2_)/5% CO_2_ for hypoxic incubation as described previously [22].

### Immunoprecipitation and immunoblotting

Immunoprecipitation (IP) and immuno-blotting assays were performed as described previously [44]. For denatured IP, cells were boiled 10 min in 2% SDS-containing Tris-buffered saline (TBS), followed by sonication and 18-fold dilution with TBS containing 1% Triton X-100. The cell lysates were immunoprecipitated with specific antibody as indicated, followed by immunoblotting with specific antibodies. The membrane was stripped using stripping buffer (200 mM Glycine, 1% SDS, pH 2.5) for re-immunoblotting.

### Immunofluorescence

Immunofluorescence assays were performed as previously described [18]. Briefly, cells were washed with ice -cold phosphate-buffered saline (PBS) and incubated on polylysine treated coverslips for 20 min (HEK293 cells were directly grow on sterile coverslips and washed once with PBS) and then fixed in 3% paraformaldehyde for 20 min at room temperature. After fixation, cells were washed three times in PBS and permeabilized in PBS containing 0.2% fish skin gelatin (G-7765, Sigma), 0.2% Triton X-100 for 5 min, and then followed the primary and secondary antibodies staining. DNA was counterstained with DAPI (4′,6′-diamidino-2-phenylindole), and Coverslips were mounted with *p*-phenylenediamine. Cells were visualized with a Fluoview FV300 (Olympus Inc., Melville, NY) confocal microscope.

### Protein expression, and in vitro pull-down assays

Overnight starter cultures (50 ml) of BL21 (DE3) transformed with plasmid expressing GST, GST-fused protein, or His-fused protein were incubated into 500 ml of Luria Broth (LB) culture medium with specific antibiotic and grown at 30°C to an optical density of about 0.6 at 600 nm. After isopropylthiogalactopyranoside (IPTG) induction (1 mM, 4 hrs at 30°C), the bacteria were collected and sonicated in lysis buffer containing 20 mM Tris-HCl pH 8.0, 100 mM NaCl, 0.5% NP40, 1 mM EDTA, 1 M DTT, 5% Sarkosyl and the protease inhibitor cocktail for use with mammalian cell extracts or in vitro translated and radiolabeled proteins. His-SUMO-1, His-SUMO-2 and His-KAP1 proteins were purified by Ni^2+^-NTA Agarose chromatography (Qiagen), and GST, GST-SIM, GST-LANA_1–329_, or GST-LANA_1–329_ΔSIM was purified by Glutathione Sepharose chromatography (Amersham Biosciences). For pull-down assay, ^35^S-methionine-labeled *in vitro*-translated proteins or cell nuclear extracts were individually incubated with the relevant His or GST-fusion proteins loaded on beads for 3 hrs at 4°C in NETN binding buffer (50 mM Tris-HCl pH 7.5, 100 mM NaCl, 10 µm ZnCl_2_, 10% glycerol, freshly supplemented with 0.1 mM Dithiothreitol (DTT) and protease inhibitor). After washing, bound proteins were eluted with SDS sample buffer and analyzed by gel electrophoresis followed by direct autoradiography and scan by PhosphoImager (Amersham Biosciences Inc.) or immunoblotting with specific antibodies.

### Protein identification by mass spectrometry

Gel slices were excised and proteins subjected to tryptic digest followed by peptide identification by lc-ms/ms using a hybrid high resolution quadrupole time-of-flight electrospray mass spectrometer. Results were analyzed suing MASCOT database search tool (Matrix Science).

### Stable RNAi-expressing cell line production and transduction

The KAP1 shRNA sequence (5′-GCATGAACCCCTTGTGCTG-3′), Sin3A shRNA sequence (5′- CAACTGCTGA GAAGGTTGATTCTGT-3′), and the control sequence (5′-TGCGTTGCTAGTACCAAC-3′, non-targeting sequence), were individually inserted into pGIPz vector according to the manufacturer's instructions (Clonetech). The pGIPz containing shRNA sequence was cotransfected with Lentivrus package expressing plasmids (Rev, VSVG and gp) into Core T cells by Calcium phosphate method to generate virus. The packaged viruses were used to individually transduce target cells (BC3, BCBL1, or HEK293) and selection by 1–4 µg/ml puromycin. The RNA interfering efficiency was assessed by western blot analysis with specific antibodies.

### Chromatin immunoprecipitation

The chromatin immunoprecipitation (ChIP) experiments were done essentially as previously described with some modifications [63], [64]. Cells (3×10^8^) were cross-linked with 1.1% (v/v) formaldehyde, 100 mM NaCl, 0.5 mM EGTA, and 50 mM Tris-HCl (pH 8.0) in growth medium at 37°C for 10 min, then at 4°C for 50 min. Formaldehyde was quenched by adding 0.05 vol 2.5 M glycine. Fixed cells were washed with PBS, incubated for 15 min in 15 ml of 10 mM Tris-HCl (pH 8.0), 10 mM EDTA, 0.5 mM EGTA, and 0.25% (v/v) Triton X-100, followed by 15 min in 15 ml of 10 mM Tris-HCl (pH 8.0), 1 mM EDTA, 0.5 mM EGTA, and 200 mM NaCl, and finally sonicated in 1 ml of 10 mM Tris-HCl (pH 8.0), 1 mM EDTA, 0.5 mM EGTA, 1% (w/v) SDS plus 1 mM PMSF, 1 µg/ml aprotonin, leupettin, and pestatin) to an average fragment size of 300–500 bp. Solubilized chromatin extracts were clarified by centrifugation at 12,000 g, and diluted to 6 OD_260_ U/ml in IP buffer [140 mM NaCl, 1% (w/v) Triton X-100, 0.1% (w/v) sodium deoxycholate, 1 mM PMSF, 100 µg/ml salmon sperm DNA, and 100 µg/ml BSA]; pre-incubated for 1 h at 4°C with 10 µl/ml 50% (v/v) protein A-Agarose (Invitrogen Life Technologies) with normal mouse/rabbit sera; reconstituted in PBS, and washed several times in IP buffer. Aliquots (600 µl) were incubated with 20 µg of each specific antibody for overnight at 4°C. Immune complexes were separated into bound and unbound complexes with protein A-agarose and cross-links were reversed by treatment at 65°C overnight. After treatment with RNase A and proteinase K, samples were extracted once with phenol/chloroform, and the DNA was precipitated with 2 volumes of ethanol plus 10 µg of glycogen as carrier (Roche). Precipitated DNA was pelleted, washed once with 70% ethanol, dried, and resuspended in 100 µl of water. The DNA was analyzed by PCR using specific primer. For sequential ChIP, a second immunoprecipitation was performed using chromatin samples eluted from the agarose beads of the first ChIP by 10 mM DTT in 37°C for 30 min twice. Relative enrichment of DNA binding was shown as a percentage of input.

### Preparation of nuclear extracts

Cells were resuspended in 4 times the volume of the pellet in NE buffer A (10 mM HEPES pH 7.9, 10 mM KCl, 1.5 mM MgCl_2_ with protease inhibitors) after cold PBS wash, and incubated on ice for 1 hour, and then transfer to precold douncer and homogenize with 25 strokes. Homogenized samples were transferred to eppendorf tubes and span at 2000 rpm for 5 mins at 4°C. Aspirate supernatant and resuspend the pellet in 2 times the volume of NE Buffer B (20 mM HEPES pH 7.9, 10% glycerol, 420 mM NaCl, 1.5 mM MgCl_2_, 0.2 mM EDTA with protease inhibitors). Incubate on ice for 30 mins and Centrifuge at 13000 rpm for 20 mins at 4°C. The supernatants were transferred to fresh eppendorf tube and add an equal volume of NE Buffer C (20 mM HEPES pH 7.9, 30% glycerol, 1.5 mM MgCl_2_, 0.2 mM EDTA with protease inhibitors). Aliquot and snap freeze samples at −80°C before use.

### In vitro DNA binding affinity assay of HIF-1α complex

Two complementary 5′-biotinylated oligonucleotides with or without HIF-1α-binding site (wt: 5′-GGATTCCAAACGTGCCCAGCG GT-3′; mut: 5′-GGATTCCAATTAATCCCAGCGGT-3′ *underline* indicates HIF-1α-binding site) were annealed to be double strands DNA and coupled to streptavidin-conjugated agarose beads. Per sample, 3 µg of biotinylated oligonucleotide was incubated with 40 ul of 50% streptavidin–conjugated agarose bead slurry (Thermo scientific) in a total volume of 100 ul of a lysis buffer comprised of 50 mM Tris-HCl, pH 8.0, 15 mM NaCl, 0.1 mM EDTA, 10% glycerol, 10 mM *N*-ethylmaleimide (NEM), 1 mM PMSF, 1 mM DTT, 1 µg/ml aprotonin, 1 µg/ml leupeptin, 1 µg/ml pepstatin for 2 h at 4C. Cell nuclear extracts (400 µg) was incubated with 40 µl of DNA-coupled Agarose beads in lysis buffer at a total volume of 500 µl for 3 hr at 4C. The precipitated complexes were washed three times with lysis buffer. Purified DNA-binding proteins were boiled in SDS sample buffer and analyzed by SDS-PAGE and immunoblotting. Equally 5% input amount of biotinylated DNA oligonucleotide were verified by dot blot.

### Luciferase assay

Luciferase reporter plasmid pRta-luc (pGL2 containing full length (1–3087) Rta promoter) was used to detect the effect of SUMO-interacting domain of LANA on viral gene expression. The luciferase reporter assays were performed as described previously [44]. TK promoter driven Renilla luciferase was used as control to normalize the transfection efficiency.

### Extraction and quantitation of KSHV episome DNA

Total DNA was extracted by lysing buffer (10 mM Tris-HCl pH 8.0, 150 mM NaCl, 10 mM EDTA, 1% SDS) followed by proteinase K digestion. Relative numbers of KSHV episomal copies were calculated by quantitative PCR (qPCR) amplification of the terminal repeats (TR) (primers see supplementary Table S2) as previously described [65]. For extracellular viral DNA, the viral particles were obtained by following the protocol for the virion purification. Bac36 DNA was used to the standards control.

### KSHV virion purification

The equal input amount of PEL cells were subjected to induction for KSHV reactivation. After induction, the supernatant of culture medium was collected and filtered through 0.45 µm filter, and viral particles were spun down at 25,000 rpm for 2 h, at 4°C. The concentrated virus was collected and used for viral DNA quantitation.

## Supporting Information

Figure S1 The levels of the truncated mutants of LANA associate with SUMO-1 and SUMO-2. (**A**), (**B**) and (**C**) HEK293 cells were individually cotransfected with expression plasmids as indicated in the figures. At 48 hr post-transfection, cell extracts were subjected to co-immunoprecipitated (co-IP) and immunoblotting (IB) as indicated in the figure. WCL, whole cell lysate.
(TIF)

Figure S2 Deletion of LANA^SIM^ reduces the SUMO-2 modification of LANA. HEK293 cells were co-transfected with expressing plasmids as indicated. At 48 hr posttransfection, cell lysates were subjected to native (*left panel*) or denature (*right panel*) immunoprecipitation (IP) with antibodies against FLAG, followed by immunoblotting analysis with antibodies as indicated. The same membrane was stripped and reblotted (reIB) with indicated antibodies. The relative density of SUMOylated LANA (suLANA) and native LANA is presented.
(TIF)

Figure S3 Hypoxic stress attenuates SUMO-2 modification of endogenous LANA in PEL cells. KSHV-positive BC3 and negative BJAB cells were individually treated with or without hypoxia (CoCl_2_ or 1% O_2_) for overnight before harvest. Cell extracts were subjected or directly immunoblotting against SUMO-2, LANA or GAPDH, or co-immunoprecipitated (co-IP) with SUMO-2 antibodies followed by immunoblotting (IB) against LANA, The relative density (RD) of SUMO-modified (suLANA) and native LANA is shown at right panel.
(TIF)

Figure S4 Lysine 1140 is critical for LANA to be SUMOylated *in vivo*. The predicted SUMOylation residues of LANA based on the consensus SUMOylation sequence ΨKxE/D is showed at the bottom panel. HEK293 cells were co-transfected with expression plasmids as indicated in the figure. At 48 hr posttransfection, whole cell lysates were subjected to immunoprecipitated (IP) followed by immunoblotting (IB) as indicated. The same membrane was stripped and reblotted (reIB) with indicated antibodies. The relative density of SUMOylated LANA (suLANA) is presented.
(TIF)

Figure S5 The LANA^SIM^-mediated inhibitory complex contributes to the self-interaction and SUMOylation of LANA. (**A**) The LANA^SIM^ plays a role in the amino- and carboxyl- terminal interaction of LANA. HEK293 cells were transfected with the expression plasmids as indicated in the figure and then harvested at 48 hr posttransfection. Whole cell lysates were subjected to co-immunoprecipitated (Co-IP) and immunoblotted (IB) as indicated. The same membrane was stripped and reblotted (reIB) with indicated antibodies. Relative density of the amino- and carboxyl-terminal interaction is shown at the bottom panel. HC, heavy chain. (**B**) C-terminal SUMOylation of LANA induced by its N-terminal domain is dependent of KAP1. Cells were co-transfected with plasmid expressing shCtrl or shKAP1 in the presence of LANA-C_842–1162_-FLAG combination with or without LANA-N_1–340_-myc. At 48 hr posttransfection, cell extracts were subjected to SUMOyaltion assays as indicated. WCL, whole cell lysate.
(TIF)

Table S1 Positions of LANA's potential SIM motifs, acidic domains and SUMOylated sites.
(DOC)

Table S2 Primers used for qPCR.
(DOC)
