# Supplementary figures and images for: A Unique SUMO-2-Interacting Motif within LANA Is Essential for KSHV Latency
Source: PLoS Pathog. 2013 Nov 21;9(11):e1003750. doi: 10.1371/journal.ppat.1003750 (PMC3836728; doi:10.1371/journal.ppat.1003750)

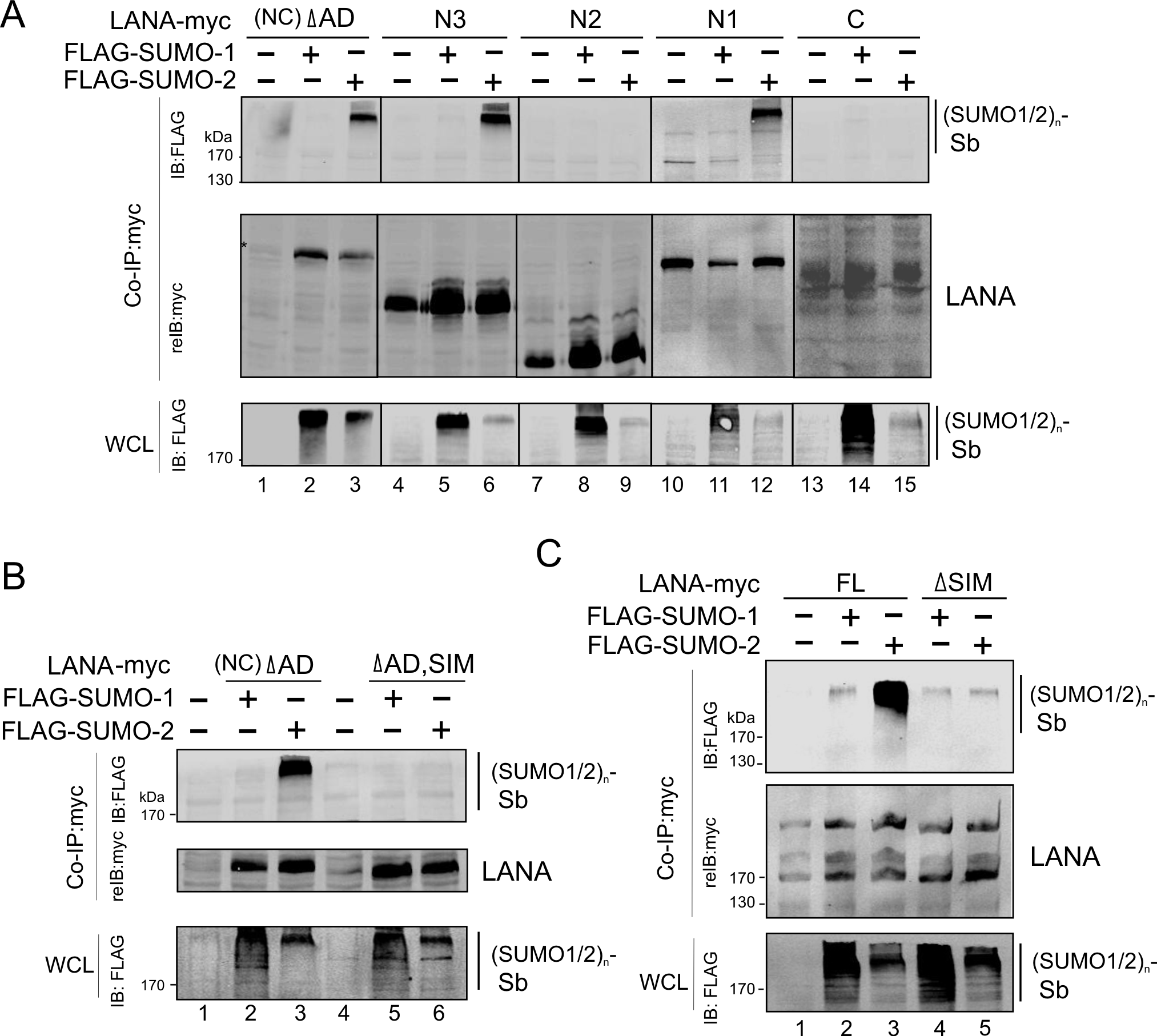

Supplement: Figure S1 — The levels of the truncated mutants of LANA associate with SUMO-1 and SUMO-2. (A), (B) and (C) HEK293 cells were individually cotransfected with expression plasmids as indicated in the figures. At 48 hr post-transfection, cell extracts were subjected to co-immunoprecipitated (co-IP) and immunoblotting (IB) as indicated in the figure. WCL, whole cell lysate. (TIF) [file ppat.1003750.s001.tif]

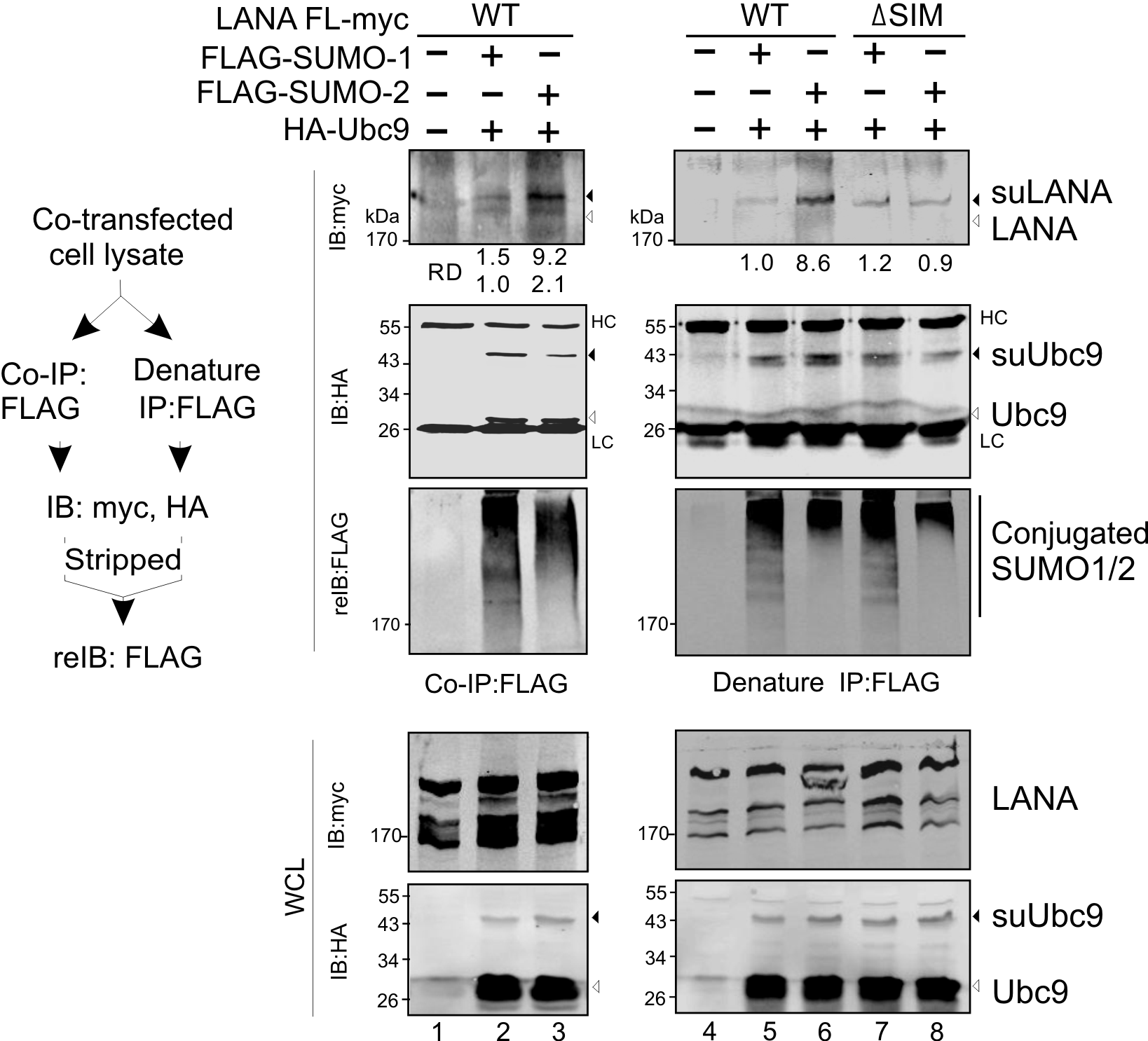

Supplement: Figure S2 — Deletion of LANASIM reduces the SUMO-2 modification of LANA. HEK293 cells were co-transfected with expressing plasmids as indicated. At 48 hr posttransfection, cell lysates were subjected to native (left panel) or denature (right panel) immunoprecipitation (IP) with antibodies against FLAG, followed by immunoblotting analysis with antibodies as indicated. The same membrane was stripped and reblotted (reIB) with indicated antibodies. The relative density of SUMOylated LANA (suLANA) and native LANA is presented. (TIF) [file ppat.1003750.s002.tif]

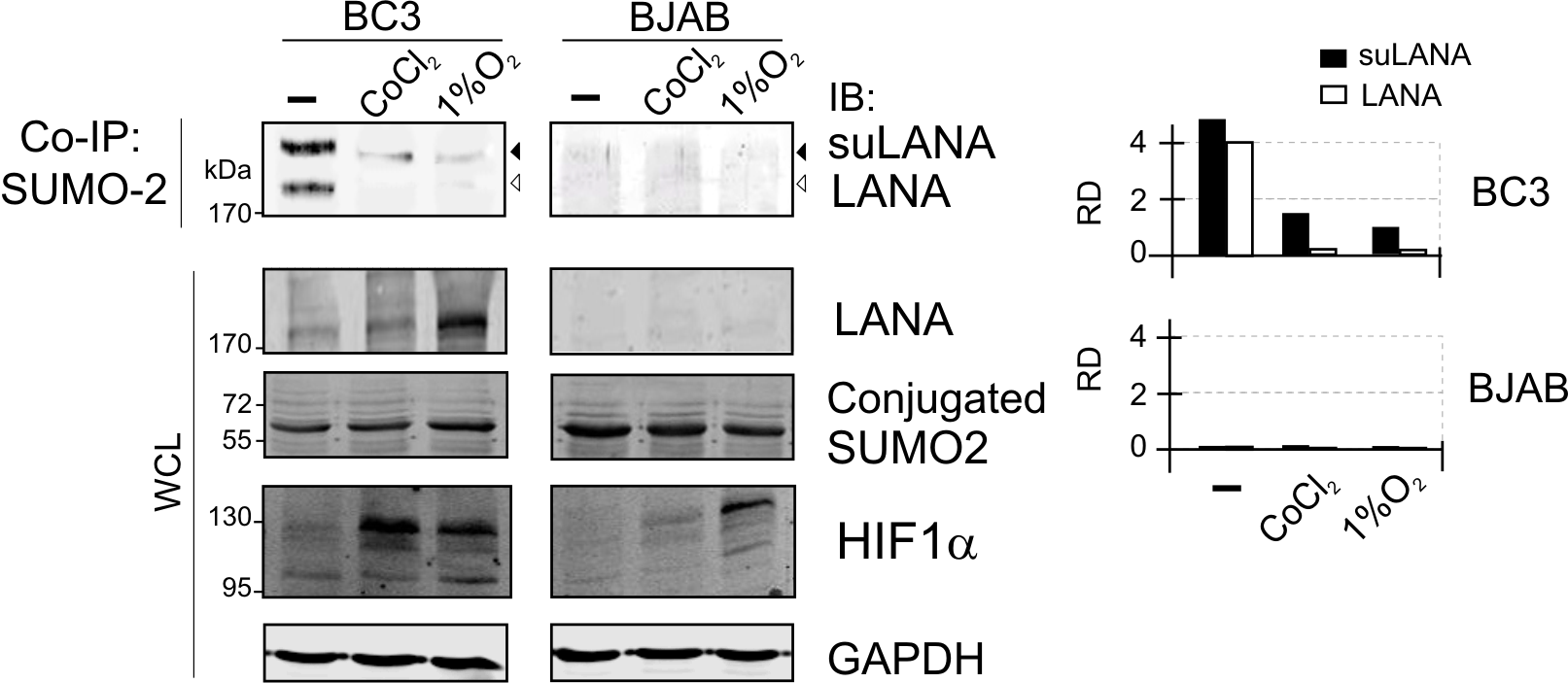

Supplement: Figure S3 — Hypoxic stress attenuates SUMO-2 modification of endogenous LANA in PEL cells. KSHV-positive BC3 and negative BJAB cells were individually treated with or without hypoxia (CoCl2 or 1% O2) for overnight before harvest. Cell extracts were subjected or directly immunoblotting against SUMO-2, LANA or GAPDH, or co-immunoprecipitated (co-IP) with SUMO-2 antibodies followed by immunoblotting (IB) against LANA, The relative density (RD) of SUMO-modified (suLANA) and native LANA is shown at right panel. (TIF) [file ppat.1003750.s003.tif]

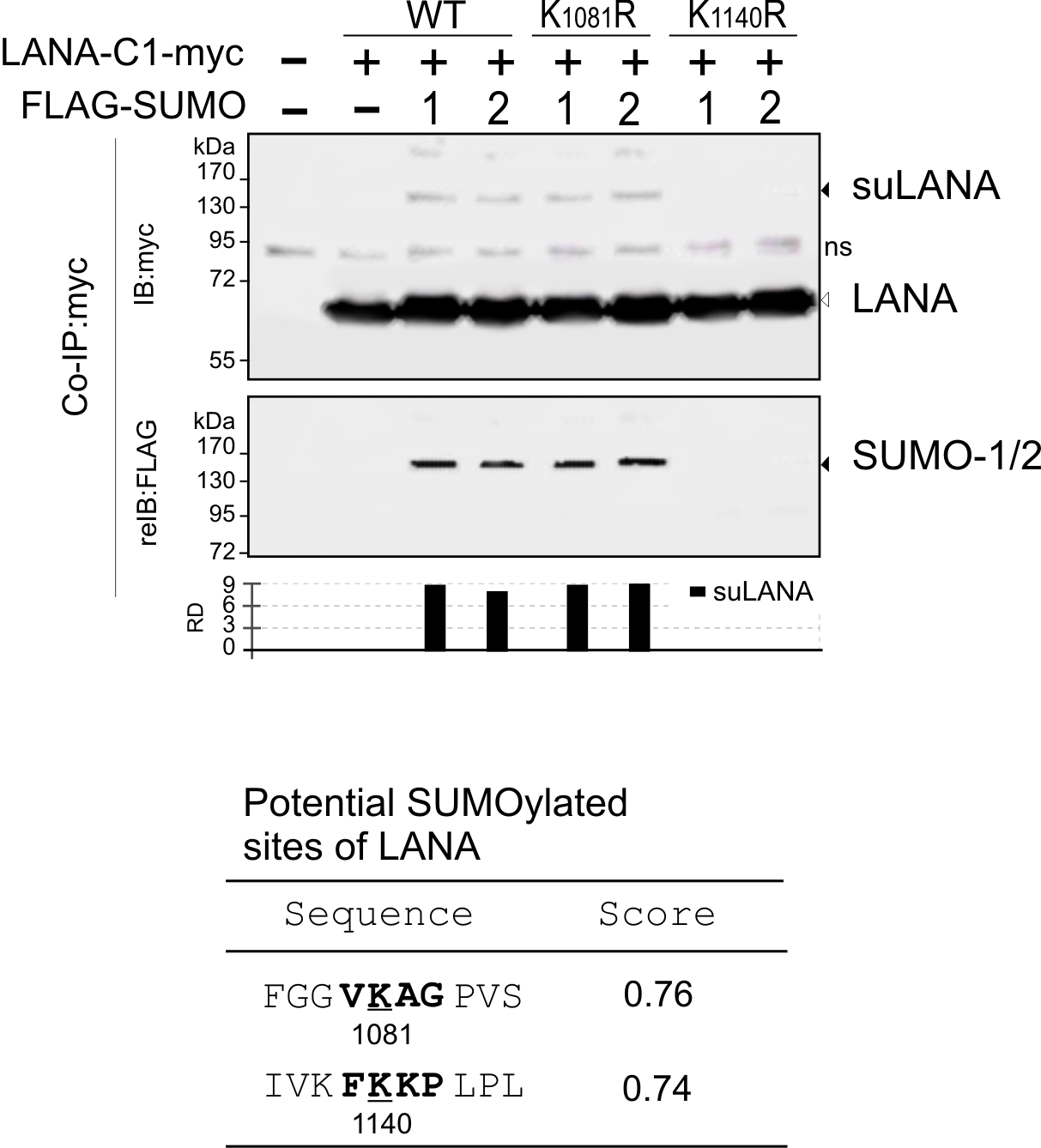

Supplement: Figure S4 — Lysine 1140 is critical for LANA to be SUMOylated in vivo. The predicted SUMOylation residues of LANA based on the consensus SUMOylation sequence ΨKxE/D is showed at the bottom panel. HEK293 cells were co-transfected with expression plasmids as indicated in the figure. At 48 hr posttransfection, whole cell lysates were subjected to immunoprecipitated (IP) followed by immunoblotting (IB) as indicated. The same membrane was stripped and reblotted (reIB) with indicated antibodies. The relative density of SUMOylated LANA (suLANA) is presented. (TIF) [file ppat.1003750.s004.tif]

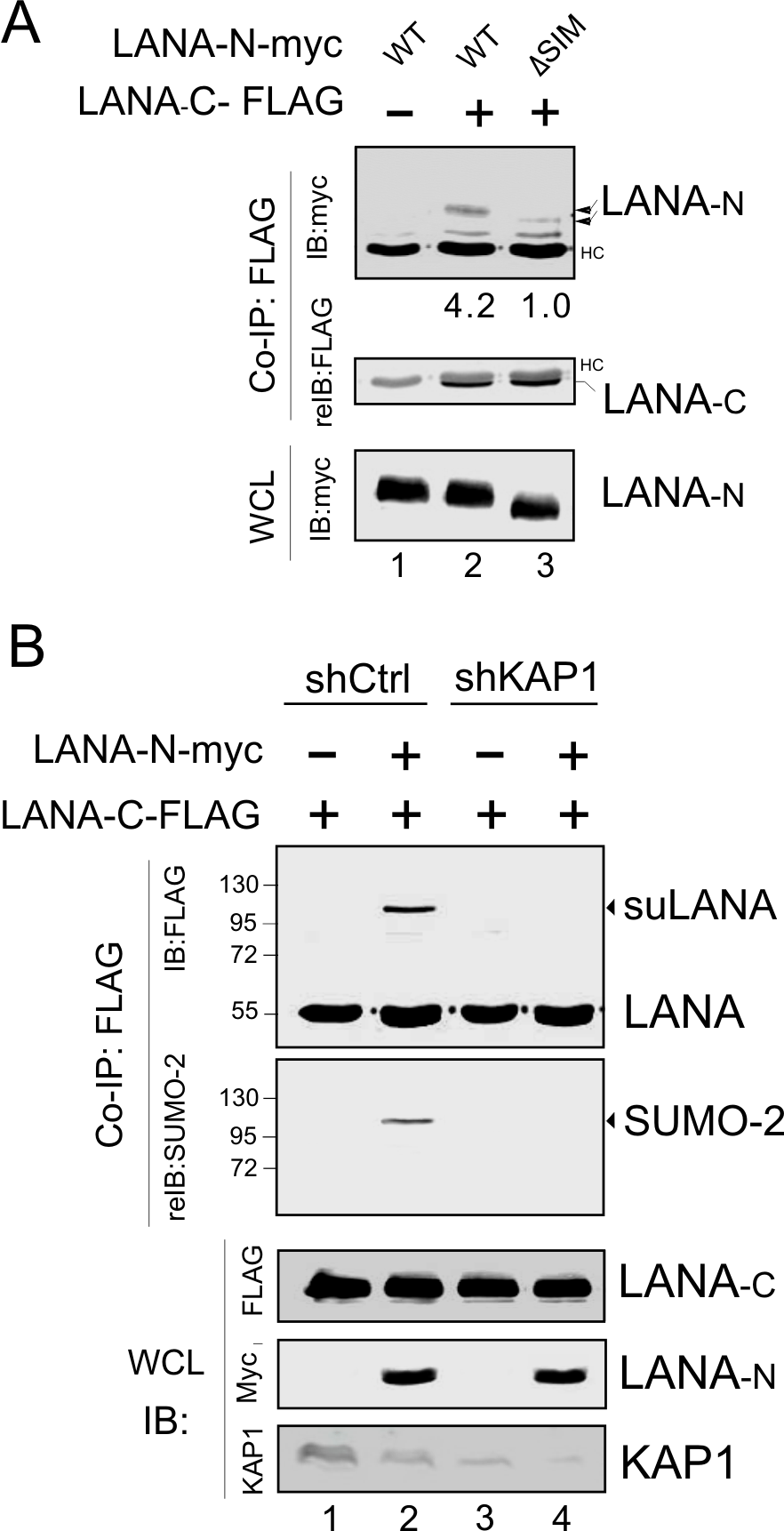

Supplement: Figure S5 — The LANASIM-mediated inhibitory complex contributes to the self-interaction and SUMOylation of LANA. (A) The LANASIM plays a role in the amino- and carboxyl- terminal interaction of LANA. HEK293 cells were transfected with the expression plasmids as indicated in the figure and then harvested at 48 hr posttransfection. Whole cell lysates were subjected to co-immunoprecipitated (Co-IP) and immunoblotted (IB) as indicated. The same membrane was stripped and reblotted (reIB) with indicated antibodies. Relative density of the amino- and carboxyl-terminal interaction is shown at the bottom panel. HC, heavy chain. (B) C-terminal SUMOylation of LANA induced by its N-terminal domain is dependent of KAP1. Cells were co-transfected with plasmid expressing shCtrl or shKAP1 in the presence of LANA-C842–1162-FLAG combination with or without LANA-N1–340-myc. At 48 hr posttransfection, cell extracts were subjected to SUMOyaltion assays as indicated. WCL, whole cell lysate. (TIF) [file ppat.1003750.s005.tif]
