# Supplementary material for: A Unique SUMO-2-Interacting Motif within LANA Is Essential for KSHV Latency
Source: PLoS Pathog. 2013 Nov 21;9(11):e1003750. doi: 10.1371/journal.ppat.1003750 (PMC3836728; doi:10.1371/journal.ppat.1003750)
Supplement: Table S1 — Positions of LANA's potential SIM motifs, acidic domains and SUMOylated sites. (DOC) [file ppat.1003750.s006.doc]

Table S1. Positions of LANA’s potential SIM motifs, acidic domains

and SUMOylated sites

MAPPGMRLRS GRSTGAPLTR GSCRKRNRSP ERCDLGDDLH LQPRRKHVAD 50

SIDGRECGPH TLPIPGSPTV FTSGLPAFVS SPTLPVAPIP SPAPATPLPP 100

PALLPPVTT***S* *SS***PIPPSHPV SPGTTDTH***SP S***PALPPTQSP ESSQRPPLSS 150

PTGRPDSSTP MRPPPSQQTT PPHSPTTPPP EPP***SKS***SPDS LAPSTLRSLR 200

KRRLSSPQGP STLNPICQSP PVSPPRCDFA NRSVYPPWAT ESP***IYV****G****SSS*** 250

LN233 **SIM1**(244-250)

DGDTPPRQPP TSP***ISI****G****SSS* PS**EGSWGDDT AMLVLLAEIA EEASKNEKEC 300

**SIM2**(264-270)

SENNQAGEDN GDNEISKESQ VDKDDNDNKD DEEEQETDEE DEEDDEEDDE 350

LN340

EDDEEDDEED DEEDDEEDDE EDDEEDDEED DEEDDEEEDE EEDEEEDEEE 400

EDEEDDDDED NEDEEDDEEE DKKEDEEDGG DGNKTLSIQS SQQQQEPQQQ 450

EPQQQEPQQQ EPLQEPQQQE PQQQEPQQQE PLQEPQQQEP QQQEPLQEPQ 500

QQEPQQQEPQ QQEPQQQEPQ QQEPQQQEPQ QQEPQQQEPQ QQEPQQQEPQ 550

QREPQQREPQ QREPQQREPQ QREPQQREPQ QREPQQREPQ QREPQQQDEQ 600

QQDEQQQDEQ QQDEQQQDEQ QQDEQQQDEQ QQDEQQQDEQ QQDEQQQDEQ 650

QQDEQQQDEQ QQDEQQQDEQ QQDEQQQDEQ QQDEQQQDEQ QQDEQQQDEQ 700

EQQDEQEQQD EQEQQDEQQQ DEQQQQDEQQ QQDEQQQQDE QQQQDEQQQQ 750

**Acidic domain (AD)**

DEQEQQEEQE QQEEQEQELE EQEQELEDQE QELEEQEQEL EEQEQELEEQ 800

LN842

EQELEEQEQE LEEQEQELEE QEQELEEQEQ ELEEQEQELE EQEVEEQEQE 850

VEEQEQEQEE QELEEVEEQE QEQEEQEEQE LEEVEEQEEQ ELEEVEEQEE 900

LN925

QELEEVEEQE QQELEEVEEQ EQQGVEQQEQ ETVEEP***IIL****H* *G****SSS***EDEMEV 950

DYPVVSTHEQ IASSPPGDNT PDDDPQPGPS REYRYVLRTS PPHRPGVRMR 1000

R***VPV***THPKKP HPRYQQPPVP YRQIDDCPAK ARPQHIFYRR FLGKDGRRDP 1050

KCQWKFAVIF WGNDPYGLKK LSQAFQFGGV **K**AGPVSCLPH PGPDQSPITY 1100

**K1081R**

C***VYV***YCQNKD TSKKVQMARL AWEASHPLAG NLQSSIVKF**K** KPLPLTQPGE 1150

**K1140R**

NQGPGDSPQE MT
