# Supplementary material for: A Unique SUMO-2-Interacting Motif within LANA Is Essential for KSHV Latency
Source: PLoS Pathog. 2013 Nov 21;9(11):e1003750. doi: 10.1371/journal.ppat.1003750 (PMC3836728; doi:10.1371/journal.ppat.1003750)
Supplement: Table S2 — Primers used for qPCR. (DOC) [file ppat.1003750.s007.doc]

Table S2. Primers used for qPCR

| Target | Primers sequences |
| --- | --- |
| GAPDH  TR | Sense Primer: 5’-ACGACCACTTTGTCAAGCTC-3’  Antisense Primer: 5’-GGTCTACATGGCAACTGTGA-3’  Sense Primer: 5’- GGGGCGCGGGGTGTTCACGTAGT -3’  Antisense Primer:5’- GGGGGCGCCCTCTCTCTACT-3’ |
| ORF50P | Sense Primer: 5’- GGGCGTGTTTTATTATTTCC -3’  Antisense Primer:5’- AGTGTCCTGGAAGAGTATGG -3’ |
